# Emerging Role of HMGB1 in the Pathogenesis of Schistosomiasis Liver Fibrosis

**DOI:** 10.3389/fimmu.2018.01979

**Published:** 2018-09-12

**Authors:** Amanda R. R. Vicentino, Vitor C. Carneiro, Diego Allonso, Rafael de Freitas Guilherme, Claudia F. Benjamim, Hílton A. M. dos Santos, Fabíola Xavier, Alexandre dos Santos Pyrrho, Juliana de Assis Silva Gomes, Matheus de Castro Fonseca, Rodrigo C. de Oliveira, Thiago A. Pereira, Leandro Ladislau, José R. Lambertucci, Marcelo R. Fantappié

**Affiliations:** ^1^Instituto de Bioquímica Médica Leopoldo de Meis, Universidade Federal do Rio de Janeiro, Rio de Janeiro, Brazil; ^2^Departamento de Biotecnologia Farmacêutica, Faculdade de Farmácia, Universidade Federal do Rio de Janeiro, Rio de Janeiro, Brazil; ^3^Departamento de Farmacologia Básica e Clínica, Universidade Federal do Rio de Janeiro, Rio de Janeiro, Brazil; ^4^Departamento de Análises Clínicas e Toxicológicas, Faculdade de Farmácia, Universidade Federal do Rio de Janeiro, Rio de Janeiro, Brazil; ^5^Departamento de Morfologia, Instituto de Ciências Biológicas, Universidade Federal de Minas Gerais, Belo Horizonte, Brazil; ^6^Laboratório Nacional de Biociências, Centro de Pesquisa em Energia e Materiais, Campinas, Brazil; ^7^Centro de Pesquisas René Rachou, Fundação Oswaldo Cruz, Belo Horizonte, Brazil; ^8^Institute for Stem Cell Biology and Regenerative Medicine, Stanford University School of Medicine, Stanford, CA, United States; ^9^Faculdade de Medicina, Universidade Federal de Minas Gerais, Belo Horizonte, Brazil

**Keywords:** schistosomiasis, HMGB1, granuloma, liver fibrosis, drugs therapy

## Abstract

In chronic schistosomiasis, liver fibrosis is linked to portal hypertension, which is a condition associated with high mortality and morbidity. High mobility group box 1 (HMGB1) was originally described as a nuclear protein that functions as a structural co-factor in transcriptional regulation. However, HMGB1 can also be secreted into the extracellular milieu under appropriate signal stimulation. Extracellular HMGB1 acts as a multifunctional cytokine that contributes to infection, injury, inflammation, and immune responses by binding to specific cell-surface receptors. HMGB1 is involved in fibrotic diseases. From a clinical perspective, HMGB1 inhibition may represent a promising therapeutic approach for treating tissue fibrosis. In this study, we demonstrate elevated levels of HMGB1 in the sera in experimental mice or in patients with schistosomiasis. Using immunohistochemistry, we demonstrated that HMGB1 trafficking in the hepatocytes of mice suffering from acute schistosomiasis was inhibited by Glycyrrhizin, a well-known HMGB1 direct inhibitor, as well as by DIC, a novel and potential anti-HMGB1 compound. HMGB1 inhibition led to significant downregulation of IL-6, IL4, IL-5, IL-13, IL-17A, which are involved in the exacerbation of the immune response and liver fibrogenesis. Importantly, infected mice that were treated with DIC or GZR to inhibit HMGB1 pro-inflammatory activity showed a significant increase in survival and a reduction of over 50% in the area of liver fibrosis. Taken together, our findings indicate that HMGB1 is a key mediator of schistosomotic granuloma formation and liver fibrosis and may represent an outstanding target for the treatment of schistosomiasis.

## Introduction

Schistosomiasis mansoni is a chronic hepatic disease caused by the trematode *S. mansoni* (1, 2). The disease remains a major public health problem, affecting millions of people in tropical countries, but it is endemic in Africa (3–5). Adult schistosomes inhabit the mesenteric veins, where they lay hundreds of eggs daily. A portion of these eggs is retained in the liver tissue. Given the high immunogenicity of the eggs, the pathology of schistosomiasis is largely attributed to the intense granulomatous inflammation and subsequent fibrosis induced by egg antigens (4).

*Schistosoma mansoni* egg-induced granuloma formation is primarily dependent on CD4^+^ T cell responses, and interleukin (IL)-4 and IL-13 are the main cytokines driving this reaction (6). Due to continuous antigenic stimulation from the trapped egg, inflammatory, and immune cells are sequentially recruited to sites of infection, leading to the formation of periovular granulomas, and chronic liver fibrosis in infected individuals. This condition results in portal hypertension and variceal bleeding, which are the primary causes of mortality from schistosomiasis (1, 3, 7–9).

Liver fibrosis represents an intense wound-healing process characterized by excessive deposition of extracellular matrix (ECM) in response to chronic injury, which is frequently driven by inflammation (10–12). Some soluble factors produced by invading inflammatory cells, such as pro-inflammatory and pro-fibrogenic cytokines and chemokines, including IL-13, play a pivotal role in the activation and transformation process of hepatic stellate cells (HSCs) (13, 14). HSCs are located in the space of Disse between the hepatocytes and the sinusoidal endothelium and play a central role in the progression of ECM deposition and liver fibrosis by the transformation of HSCs into proliferative and fibrogenic myofibroblast-like cells (6, 14–16).

High mobility group box 1 (HMGB1) was originally described as a chromosomal protein playing a role in nearly all DNA transactions (17–19). HMGB1 was later discovered also to play an extracellular role (20). HMGB1 is released from necrotic cells (21) or actively secreted under appropriate stimuli (22) and acts as a multifunctional alarmin (23–27). HMGB1, which under physiological conditions is an abundant chromosome-bound protein (18), under cell damage, can be acetylated, released from the nucleus to the cytoplasm and secreted to the extracellular milieu. Extracellular HMGB1 will bind to TLR4/9 and/or RAGE (28–30) of the host target cells. The HMGB1-receptor complex will then trigger cell activation to release inflammatory mediators. Thus, HMGB1 has a broad repertoire of immunological activities, such as induction of cytokine production, cell proliferation, chemotaxis, and differentiation (31–35).

Recently, emerging studies have indicated that HMGB1 is closely associated with fibrotic disorders, including liver fibrosis (36). A recent study confirmed that HMGB1 up-regulates alpha-smooth muscle actin (α-SMA) expression in HSCs. Moreover, HMGB1 activates HSCs and exhibits pro-fibrogenic effects on liver grafts, either by increasing the HSC population and ECM content in liver grafts or by transforming HSCs into myofibroblasts (37). HMGB1 serum levels are significantly increased in patients with liver fibrosis, which is a non-invasive, repeatable, and convenient marker of infection with the hepatitis B virus (38). Curcumin is effective in preventing liver fibrosis, partly due to downregulation of HMGB1, TLR2, and TLR4 via the inhibition of pro-inflammatory mediators and HSC activation (39). In this regard, from a clinical perspective, HMGB1 may represent a new target to better understand reparative liver injuries (36).

Synthetic compounds containing isoxazole (or isoxazoline) rings possess strong immunomodulatory activities (40–42). We previously demonstrated that a synthetic 3-chloro-5-(4-pyridyl)-4,5-dihydroisoxazole compound (named DIC) effectively decreased TNF-α and IL-6 released from LPS-stimulated macrophages. Importantly, DIC completely prevented HMGB1 nuclear translocation and inhibited the NF-kB and MAPK pathways (42). Another well-known inhibitor of HMGB1 is Glycyrrhizin (GZR), a natural triterpene found in the roots and rhizomes of licorice with anti-inflammatory properties. Glycyrrhizin binds directly to both HMG boxes of HMGB1 and inhibits its pro-inflammatory activities (43, 44).

In this work, we describe for the first time an increase in HMGB1 in the sera of mice and patients infected with *S. mansoni*. Moreover, we show that inhibition of HMGB1 by DIC culminated in healthier hepatic parenchyma with reduced fibrosis, and small and individualized granulomas in murine schistosomiasis. Importantly, DIC treatment promoted the significant survival of animals infected with *S. mansoni*. Together, our findings indicate that HMGB1 is a key mediator of schistosomotic granuloma formation and liver fibrosis and may represent a useful target for the treatment of schistosomiasis.

## Materials and methods

### Determination of HMGB1 levels in the plasma of patients with schistosomiasis

Samples from patients with acute (11 donors) and chronic schistosomiasis (100 donors) were collected on different days after the onset of symptoms and from patients experiencing primary or secondary infection. The patients were selected from Caju and São Pedro at a village endemic for *S. mansoni*, located in Minas Gerais, Brazil. Circulating HMGB1 concentrations were measured in healthy blood donors (87 donors) and donors with schistosomiasis using a quantitative capture ELISA assay (43).

### *Schistosoma mansoni* infection in mice

Four-week-old BALB/c male mice (CECAL / FIOCRUZ, Rio de Janeiro, Brazil) were housed in an animal facility with a 12:12-h light/dark cycle, with *ad libitum* water and free access to standard chow (BIOBASE, Santa Catarina, Brazil). The mice were exposed to parasites for 40 min by sitting in a water bath enriched with ~80 *S. mansoni* cercariae (strain BH), which were kindly provided by Dr. Silvana Thiengo (Laboratório de Malacologia, FIOCRUZ, Rio de Janeiro, Brazil).

### Experimental design

The 3-chloro-5-(4-pyridyl)-4,5-dihydroisoxazole (DIC) compound was synthesized as described in our previous work (42). Glycyrrhizin (Glycyrrhizic acid ammonium) (GZR) was purchased from Sigma (Sigma Aldrich, Missouri USA), and DIC was suspended in 4% of propylene glycol (Sigma Aldrich, Missouri USA), in 1X PBS. Treatment with GZR, DIC (10 mg/kg), or the vehicle was started 21 days post-infection and continued until the end of the experiment at 56 or 112 days post-infection for comparisons of different times of infection. The experiment was divided into 5 groups: uninfected mice (UI), infected, and untreated mice (IUT), infected mice administered the vehicle (VEH) and infected mice treated with GZR or DIC.

### Parasitological parameters

The intestines were digested as described previously by Cheever (45). Briefly, tissues were maintained in 4% KOH at room temperature for ~12 h, followed by 1 h of incubation at 37°C. The results are expressed as eggs per gram of intestine. Eight independent samples were counted.

### Estimation of hepatic enzymes activities

Enzymatic assays for the detection of serum alanine aminotransferase (ALT) and aspartate aminotransferase (AST) (Enzipharma, Rio de Janeiro, Brazil) were performed according to the manufacturer's recommendations using the plate reader SpectraMax M5®.

### Immunohistochemistry

Immunohistochemical staining (IHC) for histological sections of hepatic tissue (right lobe of the liver) was performed using monoclonal anti-HMGB1 (Abcam, Cambridge, UK, Ab79823), anti-collagen 1α (anti-Col1α-Ab34710), anti-collagen 3 (anti-Col3-Ab7778), and anti-alpha smooth muscle actin (anti-αSMA-Ab15734) antibodies. Sections of the right liver lobe were collected, fixed in 4% buffered para-formaldehyde solution (Sigma-Aldrich, Missouri, USA) and embedded in paraffin. Briefly, paraffin-embedded sections were de-waxed and hydrated and the antigen retrieval was performed by heating the sections with sodium citrate buffer (10 mM sodium citrate, 0.05% Tween 20, pH 6.0–Merck, Germany) for 30 min at 95°C. After endogenous peroxidases were inhibited in the sections (3% H_2_O_2_ in methanol for 30 min–Sigma Aldrich, Missouri, USA), the sections were incubated for 1 h with blocking buffer containing 3% BSA in 1x PBS. The primary monoclonal antibodies were 1/100 diluted in 1% BSA in 0.25% PBS-T and were applied to the sections and incubated overnight at 4°C in a humid chamber. Horseradish peroxidase labeled secondary anti-rabbit (KPL, Maryland, USA) antibody (1/100 dilution) was applied to the sections and incubated for 2 h at room temperature. The presence of proteins of interest was visualized by DAB staining (Agilent, California, USA). The sections were counterstained with hematoxylin (Sigma-Aldrich, Missouri, USA). Entellan (Merck, New Jersey, USA) was used as a mounting medium for cover slips. Bright-field pictures were acquired using a digital camera (Leica, Wetzlar, Germany) fitted on an Olympus model microscope. As negative control, liver sections were incubated with blocking buffer instead of monoclonal antibodies and then with secondary anti-rabbit antibodies for all analyses (Figure S1).

### Preparation of soluble egg antigens (SEA)

*Schistosoma mansoni* soluble egg antigens (SEA) were prepared with eggs taken from mice 8 weeks after they had been infected. Livers were removed and kept in saline solution (1.7%) at 4°C overnight in order to better isolate eggs. Eggs were harvested from the homogenized livers by differential centrifugation, after clarification of the homogenate. The absence of contaminating mice tissue fragments in the egg preparation was checked by microscopic analysis. Purified eggs were homogenized on ice in PBS with Polymyxin B (15 μg/mL) to neutralize lipopolysaccharide contamination. After repeated freezing and thawing, the homogenate was centrifuged at 14,000 *g*, 4°C for 20 min, and the supernatant was used as *S. mansoni* soluble egg antigen.

### Cytokine analysis

A Cytometric Bead Array (CBA) Mouse Th1/Th2/Th17 Cytokine Kit® (BD Company, California, USA) was used to detect the expression levels of IL-10, IL-17A, TNF-α, IFN-γ, IL-6, and IL-4 in the serum of all groups following the manufacturer's instructions. Six standard curves were obtained from one set of calibrators. Flow cytometry LSR II (BD Company, California, USA) was used to read the data, and cytokine concentrations were calculated according to the standard curve. Enzyme-linked immunosorbent assays (ELISA) for IL-5 and IL-13 were performed using specific ELISA-kits (IL-5 and 13, Peprotech, New Jersey, USA) to detect the presence of these cytokine in mouse serum obtained after euthanasia. According to the manufacturers' guidelines, standard curves were prepared to calculate protein concentrations from standard optical densities vs. concentrations. Protein levels were expressed as pg/mL ± standard errors of the mean (SEMs) of two technical replicates. A modified ELISA for HMGB1 determination was established in our laboratory and was described previously (46). The standard curve calculation was performed using the mass value of the serial dilution of the rHMGB1 (47) protein against its respective optical density (O.D.) measurement. The calculated concentrations were consistent with the reported values of commercially available HMGB1 quantification kits.

### Histopathology

Sections of the right liver lobe were collected, fixed in 4% buffered para-formaldehyde solution (Sigma-Aldrich, Missouri, USA) and embedded in paraffin. Five micrometer sections of fixed liver slices were stained with hematoxylin and eosin (H&E) and were read by bright field microscopy. The areas of hepatic granulomas were determined in histological sections from 200 granulomas per group containing central viable eggs that were randomly chosen. The granuloma areas were manually delimited in H&E images using ImageJ software (NIH, Maryland, USA). The collagen content in the liver slices was assessed and quantified by Masson Trichome and Sirius red staining. The stained images obtained from separate fields of the samples (*n* = 5) were analyzed by using ImageJ. All images were captured by a digital camera (Leica, Wetzlar, Germany) using bright field microscopy. All evaluations were performed by two different observers in a blind fashion.

### Determination of IL-13, TGF-β, Col-1, and Col-3 levels in liver extracts

Protein extracts were obtained by homogenizing a piece of the right lobe of the liver in lysis buffer containing 0.32 M sucrose, 1 mM EDTA, 1 mM EGTA, 10 mM Tris pH 7.4 and protease inhibitor cocktail (Sigma-Aldrich, Missouri, USA) in a proportion of 100 mg of tissue per 1 mL of lysis buffer. After 30 min on ice, the samples were centrifuged at 4°C for 20 min at 20,000 *g*, to pellet the cellular *debris*, followed by a quick freeze of the supernatants. Protein concentration was determined using the Bradford (Sigma-Aldrich, Missouri, USA) protein assay reagent according to the manufacturer's instructions. All samples of liver extracts were diluted 20x for determination of IL-13 (Peprotech), TGF-β (R&D System, Minneapolis, USA), Col-1 and Col-3 (Cloud-Clone, Houston, USA) by ELISA.

### Statistical analysis

The results are expressed as the mean ± SEM. Differences between groups were assessed using the ANOVA Tukey's multiple comparisons analysis. *P*-values for each comparison are available in Table S3. Animal survival data were evaluated using the Kaplan-Meier method and were compared with the log-rank test. *P* < 0.05 was considered to be statistically significant.

## Results

### HMGB1 plasma levels are increased in patients with acute and chronic schistosomiasis

**Figure 1:**
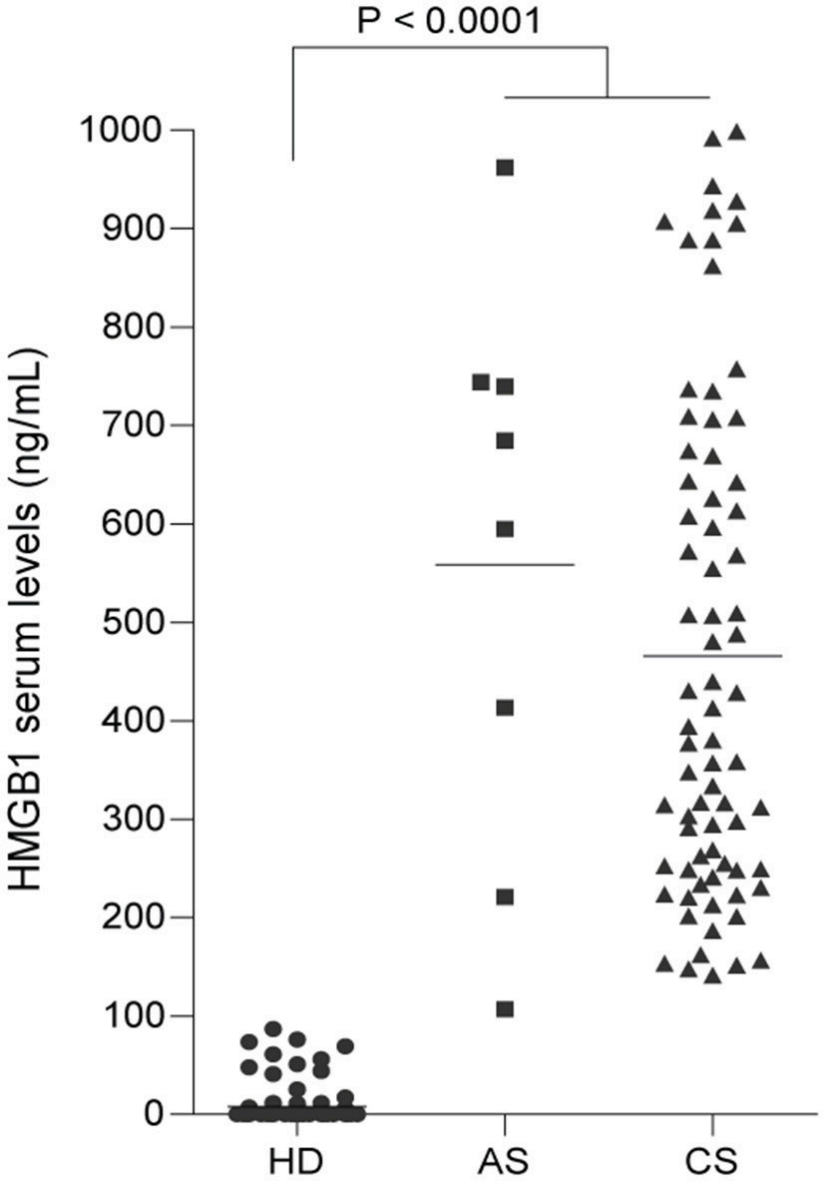
HMGB1 plasma levels are increased in patients with acute and chronic schistosomiasis. Significantly higher levels of HMGB1 were found in the sera of patients with acute (AS) or chronic schistosomiasis (CS) than in healthy donors (HD). Data are presented as the mean with SEM of 87 individuals from the HD group, 11 from the AS group and 100 from the CS group. All values were tested by ANOVA Tukey's multiple comparisons test. *P* < 0.0001 was considered statistically significant (*P* < 0.0001 HD *vs*. AS or CS).

The presence of HMGB1 in human sera is implicated in the pathogenesis of many acute and chronic inflammatory conditions (26, 48–50). Thus, we quantified the levels of HMGB1 in the sera of human patients suffering from acute or chronic schistosomiasis. Importantly, high levels of HMGB1 were detected in the plasma of both acute and chronic schistosomotic patients when compared with the sera of healthy donors (Figure 1; Table S1).

### Pathophysiological parameters of schistosomiasis

**Figure 2:**
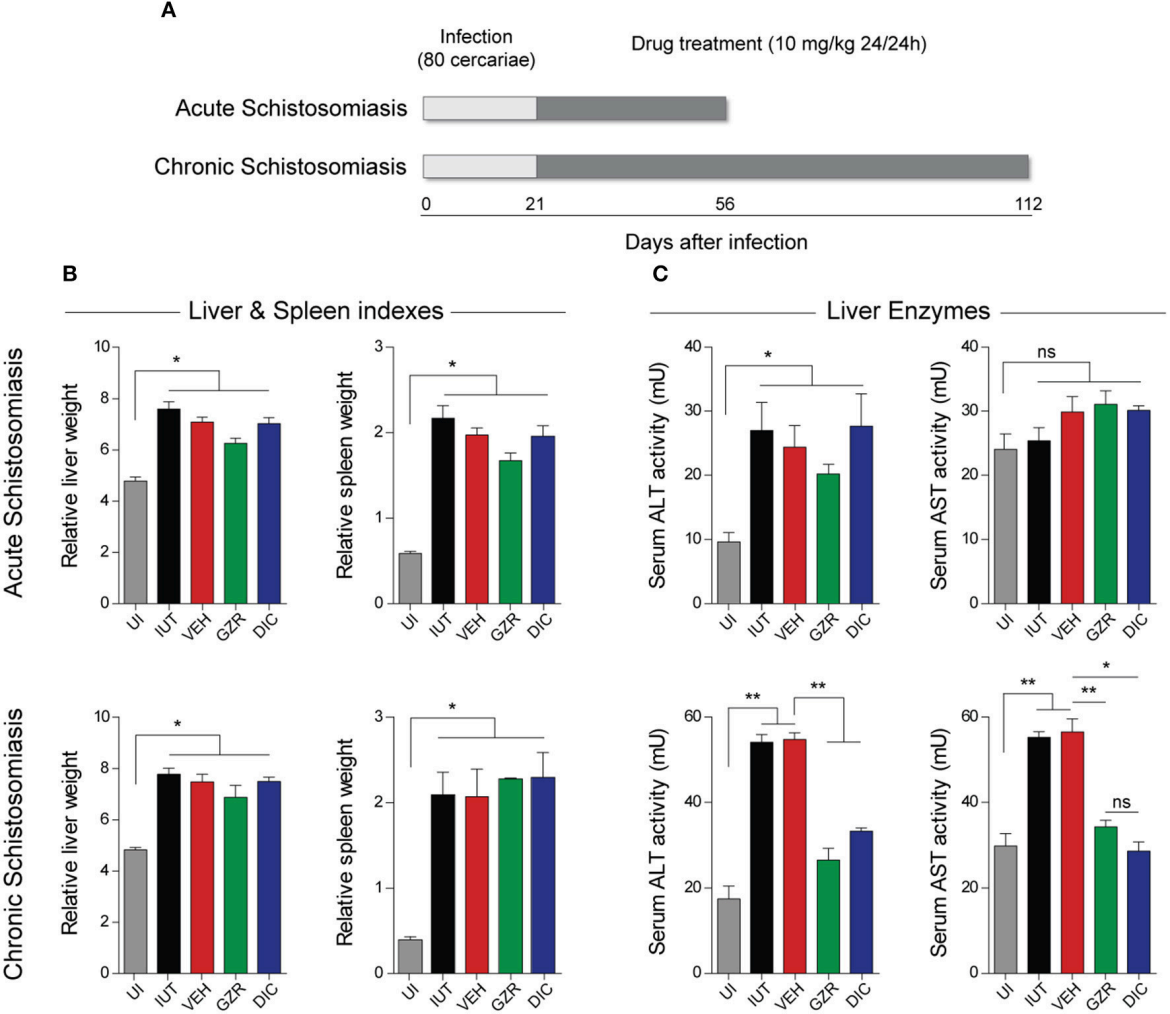
Experimental design, relative organs weights and liver enzyme activities. **(A)** BALB/c mice were infected with 80 *S. mansoni* cercariae. Mice with acute or chronic schistosomiasis were treated with daily doses of vehicle (4% propylene-glycol), or anti-HMGB1 inhibitors, Glycyrrhizin (GZR) and 3-chloro-5-(4-pyridyl)-4,5-dihydroisoxazole (DIC), both at a dose of 10 mg/kg, starting on day 21 post-infection and proceeding until day 56 in the case of acute schistosomiasis or day 112 in chronic schistosomiasis. Mice were divided into five groups as follows: uninfected mice (UI), infected and untreated mice (IUT), infected mice that received vehicle (VEH), GZR or DIC. The analyses were conducted with 40 animals per group for acute schistosomiasis and 15 animals per group for chronic schistosomiasis. **(B)** Measurements of the relative weight of the organs in acute and chronic schistosomiasis. The relative weights were calculated with the following equation: Relative organ weight = (absolute organ weight / body weight) × 100. **(C)** Determination of the activities of alanine aminotransferase (ALT) and aspartate amino transferase (AST) in the sera of acute and chronic schistosomiasis. All results (from 40 animals for acute and 15 for chronic infections) are presented as the mean ± SEM and were tested by ANOVA Tukey's multiple comparisons test. *P < 0.05 or **P < 0.0001 was considered statistically significant; ns—not significant. The *P*-values for all comparisons are available in Table S3.

To investigate the role of HMGB1 during the course of schistosomiasis in a more controlled fashion, we infected BALB/c mice with 80 *S. mansoni* cercariae for 56 and 112 days to establish an acute and chronic disease, respectively (Figure 2A). A direct involvement of HMGB1 signaling in the pathogenesis of schistosomiasis could be evoked with the use of a well-known HMGB1 direct inhibitor, Glycyrrhizin (GZR) (43). In addition, and importantly, a potential novel HMGB1 inhibitor, the 3-chloro-5-(4-pyridyl)-4,5-dihydroisoxazole (DIC) was tested in the model of schistosomiasis. We have previously shown that, *in vitro*, DIC was able to inhibit the secretion of HMGB1 from macrophages through the NF-kb and MAPK pathways (42). In this work, we compared the HMGB1 inhibitory effect of DIC with that of GZR in animals infected with *S. mansoni*. For the evaluation of toxicity, non-infected animals were treated with DIC for 56 and 112 days (mimicking the times of acute and chronic schistosomiasis; Table S2). Of note, the prolonged treatment of DIC did not alter the relative liver or spleen weights, the level of creatinine or the activity of hepatic enzymes (Table S2).

To evaluate the effect of HMGB1 inhibition on *S. mansoni*-induced hepatomegaly and splenomegaly, livers, and spleens were excised from mice after euthanasia and were weighed and the relative organ-to-body weight percentages were calculated. Mice that experienced the acute or chronic phases of the disease developed hepatosplenomegaly characteristic of schistosomiasis, regardless the treatments of GZR or DIC (Figure 2B). In addition, when we analyzed the intestinal egg loads of all groups of mice (Figure S2), no significant differences were observed, suggesting that neither GZR nor DIC has schistosomicidal activity. In fact, when we cultured adult worms (*in vitro*) in the presence of DIC, the viability of the worms was not affected (Figure S3). To evaluate the ameliorative effect of DIC treatment on the liver pathology induced by *S. mansoni* infection, the activities of ALT and AST were measured in the sera at 56 or 112 weeks post-infection. The activities of ALT and AST were significantly increased in all groups of mice with acute schistosomiasis (Figure 2C). Importantly, in mice with chronic schistosomiasis that were treated with GZR or DIC, ALT, and AST activities were significantly decreased (Figure 2C). For GZR, this result is not surprising since it is already known that this compound is hepatoprotective (43, 44).

### HMGB1 behaves differentially between acute and chronic schistosomiasis

**Figure 3:**
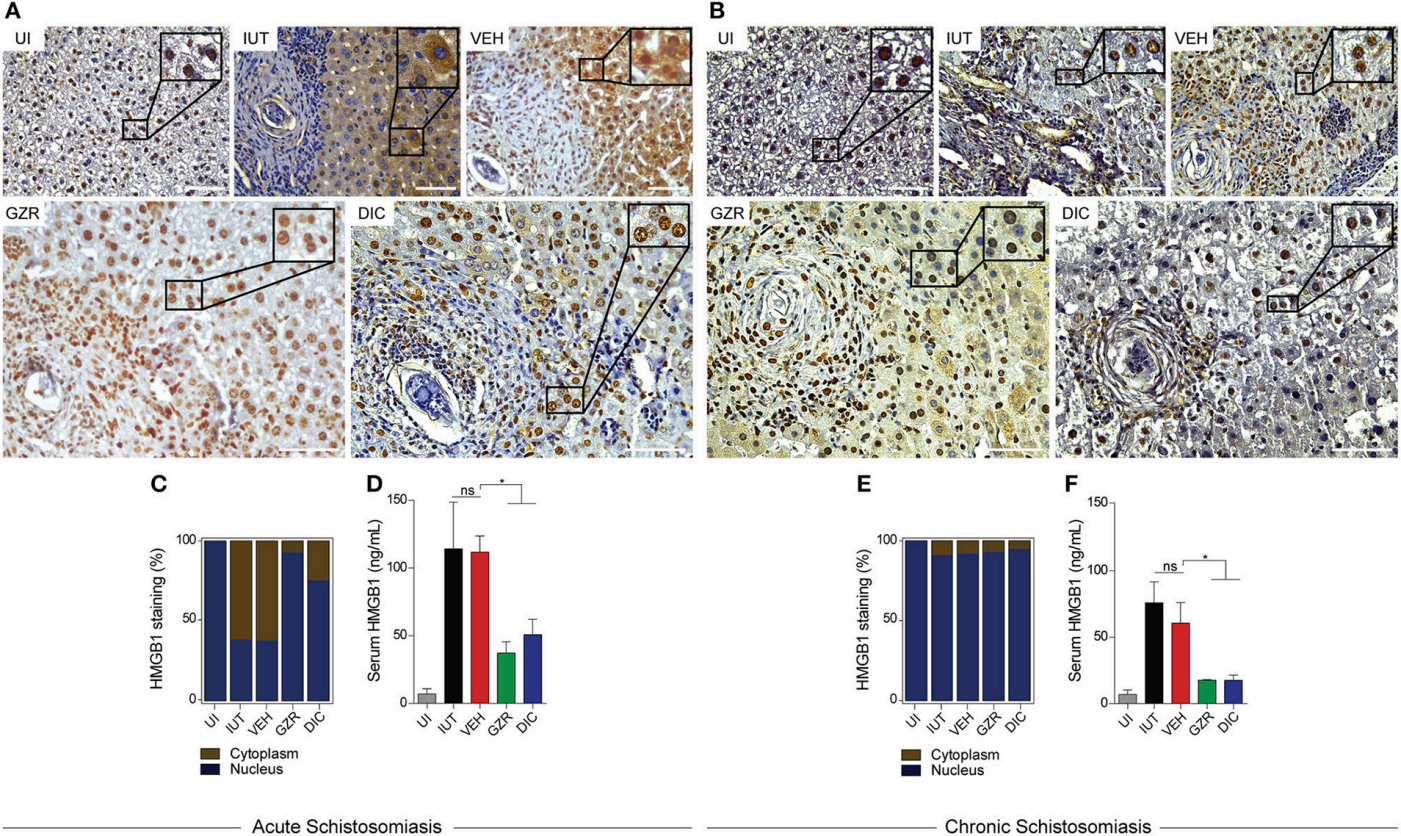
Secretion of HMGB1 is restricted in the acute phase of schistosomiasis. **(A,B)** Representative photographs of immunohistochemistry (from 5 animals per group) of HMGB1 in hepatic tissues after acute or chronic schistosomiasis. Insets show nuclear or cytoplasmic HMGB1 in an amplified manner. Photographs presented at 40x magnification. Scale bar = 50 μm. Note that both inhibitors prevented HMGB1 secretion only in the acute schistosomiasis (**A**, GZR and DIC). **(C,E)** Quantification of the immunohistochemistry, showing the percentage of hepatocytes with HMGB1 in the nucleus (blue) or cytoplasm (brown). **(D,F)** Sera levels of HMGB1 of mice with acute (40 animals) or chronic (15 animals) schistosomiasis. The results were presented as the mean ± SEM and were tested by ANOVA Tukey's multiple comparisons test. *P < 0.05 was considered statistically significant; ns—not significant. The *P*-values for all comparisons are available in Table S3. Legends of the experimental groups are as depicted in Figure 2.

Immunostaining of livers from the uninfected (UI) group revealed that HMGB1 was localized predominantly in the nucleus of hepatocytes (Figures 3A,B). Alternatively, after 56 days of infection, which characterizes the acute phase of schistosomiasis, an increased HMGB1 immunoreactivity was observed in the cytoplasm of hepatocytes around the granulomas of the infected untreated (IUT) and vehicle (VEH) control groups (Figures 3A,C). In mice that were treated with HMGB1 inhibitors (GZR or DIC), HMGB1 translocation from the nucleus to the cytoplasm was reduced, as shown by a significant decrease of staining in the cytoplasm of hepatocytes (Figures 3A,C). The specificity of the immunoreactivity of HMGB1 was confirmed by the lack of immunostaining using the secondary anti-rabbit control (Figure S1).

If our hypothesis that HMGB1 is secreted due to infection were correct, we would then expect to identify high levels of the protein in the sera of infected mice. Thus, mice with acute schistosomiasis (IUT or VEH groups) exhibited high levels of HMGB1 in their plasma, ~15-fold higher than that of uninfected mice (Figure 3D). Importantly, GZR or DIC groups exhibited a significant reduction (~50%) in the levels of HMGB1 in their sera (Figure 3D). These data confirm the effect of GRZ and DIC in inhibiting the cellular trafficking of HMGB1, as previously shown *in vitro* (42). When we analyzed the HMGB1 trafficking profile in chronically infected mice, the protein was detected mainly in the nuclei of hepatocytes (Figures 3B,E), independent of treatments. When we analyzed the levels of HMGB1 in the sera of chronically infected mice, we observed high levels of the protein in IUT and VEH groups (Figure 3F), although the levels were considerably reduced compared with the levels observed in acute infection. Animals that were treated with GZR or DIC revealed significant lower levels of HMGB1 in their sera when compared to those of control groups (Figure 3F).

To further confirm that HMGB1 indeed responds to egg-induced inflammation, we performed *in vitro* culture of primary hepatocytes treated with soluble-egg antigens (SEA) and looked at the trafficking of HMGB1. Our immunofluorescence analysis showed that SEA was able to promote HMGB1 secretion (Figure S4A, panel SEA; note high levels of HMGB1 in the cytoplasm of the cells). GZR or DIC treatments inhibited the exit of HMGB1 from the nucleus to the cytoplasm (Figure S4A, panel SEA + GZR/DIC; note the complete retention of HMGB1 in the nuclei). Therefore, our *in vitro* immunofluorescence data agree with our *in vivo* immunohistochemistry data. In addition, we showed by Western blot analysis that higher levels of extracellular HMGB1 was observed in the supernatant of the hepatocyte culture stimulated with SEA, when compared with unstimulated cells (Figure S4C). Importantly, the hepatocytes that were stimulated with SEA and treated with GZR or DIC revealed significant reductions in the levels of extracellular HMGB1 (Figure S4C). The cell viability of the hepatocytes under SEA stimuli and treatment was >80% (Figure S4D). The small amount of the hepatocyte deaths (>20%) could be explained by the stress of the cells under antigen presentation.

### HMGB1 participates in the modulation of key cytokines that are involved in granuloma formation and liver fibrosis

**Figure 4:**
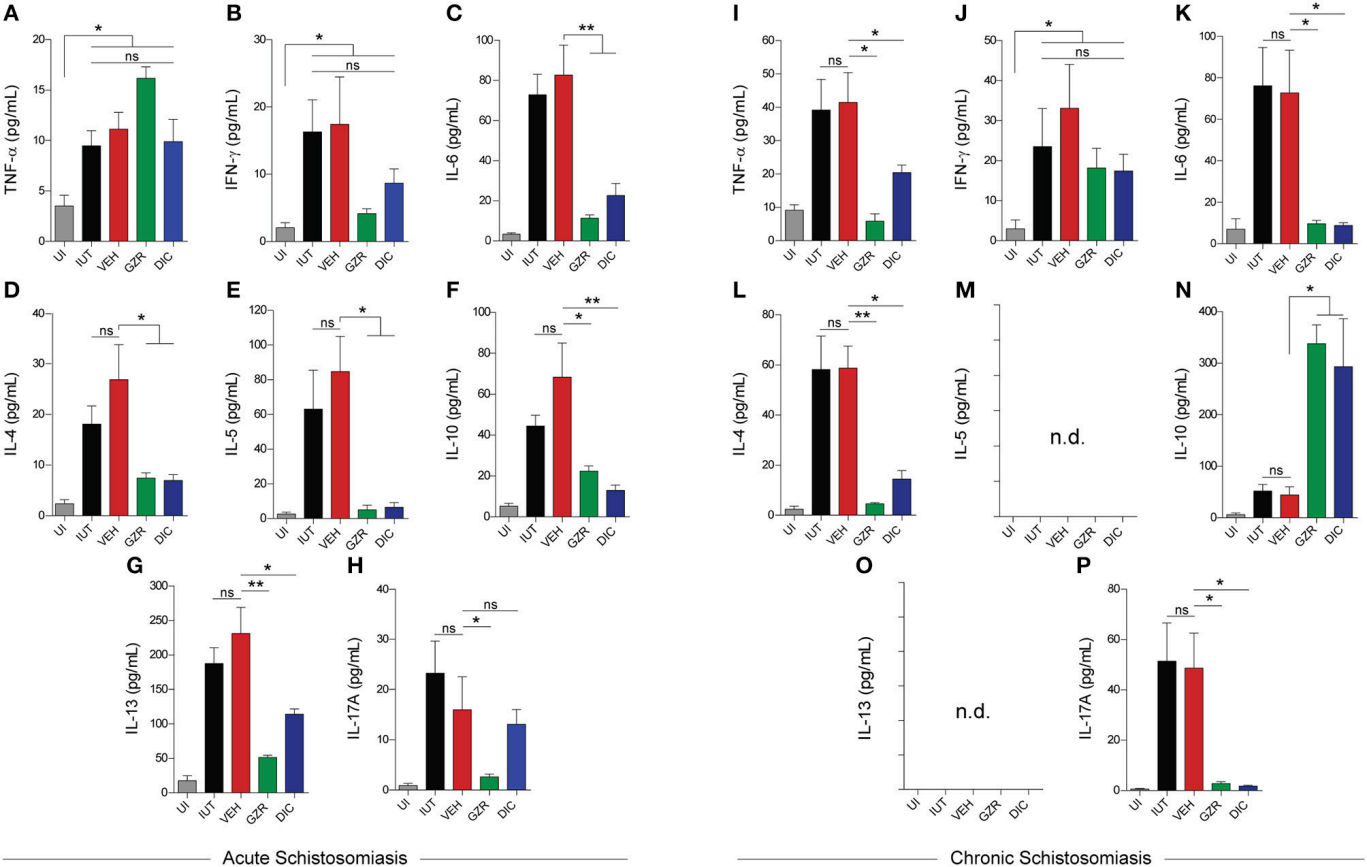
HMGB1 modulates the inflammatory responses of key cytokines involved in granuloma formation and liver fibrosis. **(A–P)** Quantification of Th1/Th2/Th17 cytokines in the sera of mice with acute and chronic schistosomiasis. In the chronic phase of schistosomiasis, IL-5 and IL-13 were not detected (n.d.). The results (from 40 animals for acute and 15 for chronic infections) were presented as the mean ± SEM and were tested by ANOVA Tukey's multiple comparisons test. *P < 0.05 or **P < 0.0001 was considered statistically significant; ns—nt significant. The *P*-values for all comparisons are available in Table S3. Legends of the experimental groups are as depicted in Figure 2.

Because cytokine dysregulation is associated with increased mortality during schistosomiasis (51), we investigated whether GZR- or DIC-mediated inhibition of HMGB1 trafficking interfered with the cytokine expression profile during acute or chronic schistosomiasis. The cytokine levels of TNF-α, IFN-γ, IL-6, IL-4, IL-5, IL-10, IL-13, and IL-17A in the sera of acute phase schistosomiasis were all significantly increased in the IUT and VEH groups compared with the UI group (Figures 4A-H). However, the cytokine levels in the sera of animals treated with GZR or DIC were markedly decreased for IL-6, IL-4, IL-5, IL-10, and IL-13 (Figures 4C-G). Of note, the levels of IL-17A were only reduced in the sera of animals treated with GZR (Figures 4H). However, during the chronic phase of schistosomiasis, the levels of TNF-α, IFN-γ, IL-6, IL-4, IL-10, and IL-17A remained increased in the IUT and VEH groups (Figures 4 I–L,N,P). As expected (52, 53), IL-5, and IL-13 levels were not detected during the chronic phase of schistosomiasis (Figures 4M,O). Importantly, inhibition of HMGB1 led to a significant increase in the anti-inflammatory cytokine IL-10 (Figure 4N). In addition, in the sera of animals treated with GZR or DIC, the levels of TNF-α, IL-6, IL-4, and IL-17A were significantly decreased (Figures 4 I,K,L,P).

### Granuloma area reduction by HMGB1 inhibition

**Figure 5:**
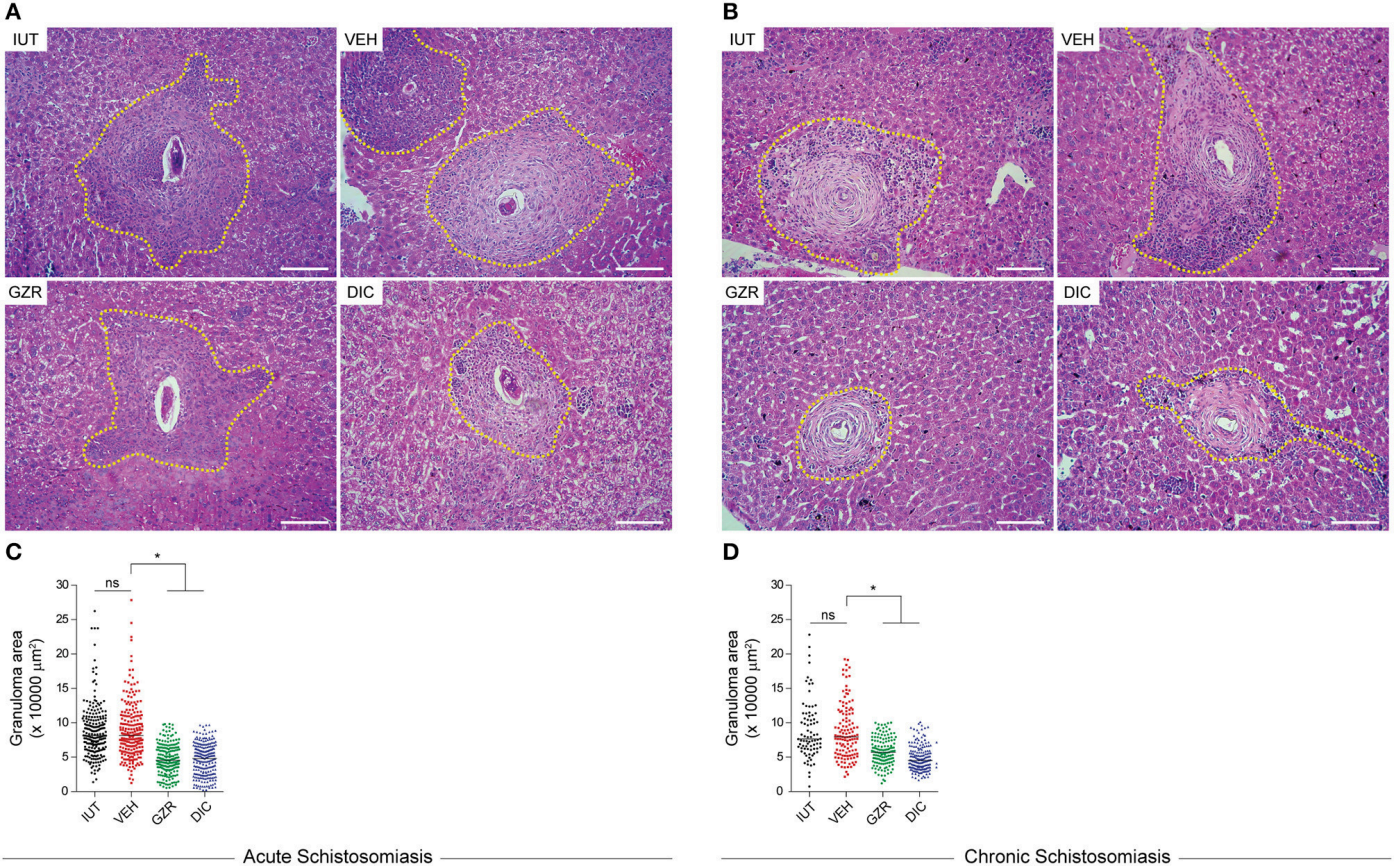
Inhibition of HMGB1 reduces schistosomotic granuloma size. **(A,B)** Representative photographs of hematoxylin and eosin-stained sections of livers. Yellow dashed lines depict the granuloma sizes. Photographs are at a magnification of 20x. Scale bars = 20 μm. **(C,D)** Quantification of the granuloma areas in the livers. Two hundred granulomas were measured for each group in acute schistosomiasis. Of note, in chronic schistosomiasis, only 50 isolated granulomas were identified and measured in IUT and VEH groups. The granuloma areas were measured using ImageJ software. The results were presented as the mean ± SEM and were tested by ANOVA Tukey's multiple comparisons test. *P < 0.0001 was considered statistically significant; ns—not significant. The *P*-values for all comparisons are available in the Table S3. Legends of the experimental groups are as depicted in Figure 2.

To investigate the role of HMGB1 down-regulation on granulomatous inflammation, hepatic granuloma areas of animals that were treated with GZR or DIC were measured at 56 or 112 days post-infection. Morphometric analysis of liver slices demonstrated a significant reduction in granuloma size after GZR or DIC treatments during both the acute and chronic phases of schistosomiasis (Figures 5A,B). Quantification of the granuloma areas revealed a reduction of 50% in the livers of animals treated with GZR or DIC (Figures 5C,D). Given that HMGB1 is involved in cell recruitment (32, 34), our data suggest a possible role of HMGB1 in egg-induced granuloma formation. It is worth noting that in the chronic phase of schistosomiasis, significantly fewer isolated granulomas were observed in the IUT and VEH groups (Figure 5D). We believe that it was difficult to identify isolated granulomas given the extensive fibrotic areas distributed across the hepatic parenchyma. In mice where the secretion of HMGB1 was inhibited, significantly more isolated granulomas were noted, and these granulomas were smaller (Figure 5D).

### HMGB1 is involved in egg-induced liver fibrogenesis

**Figure 6:**
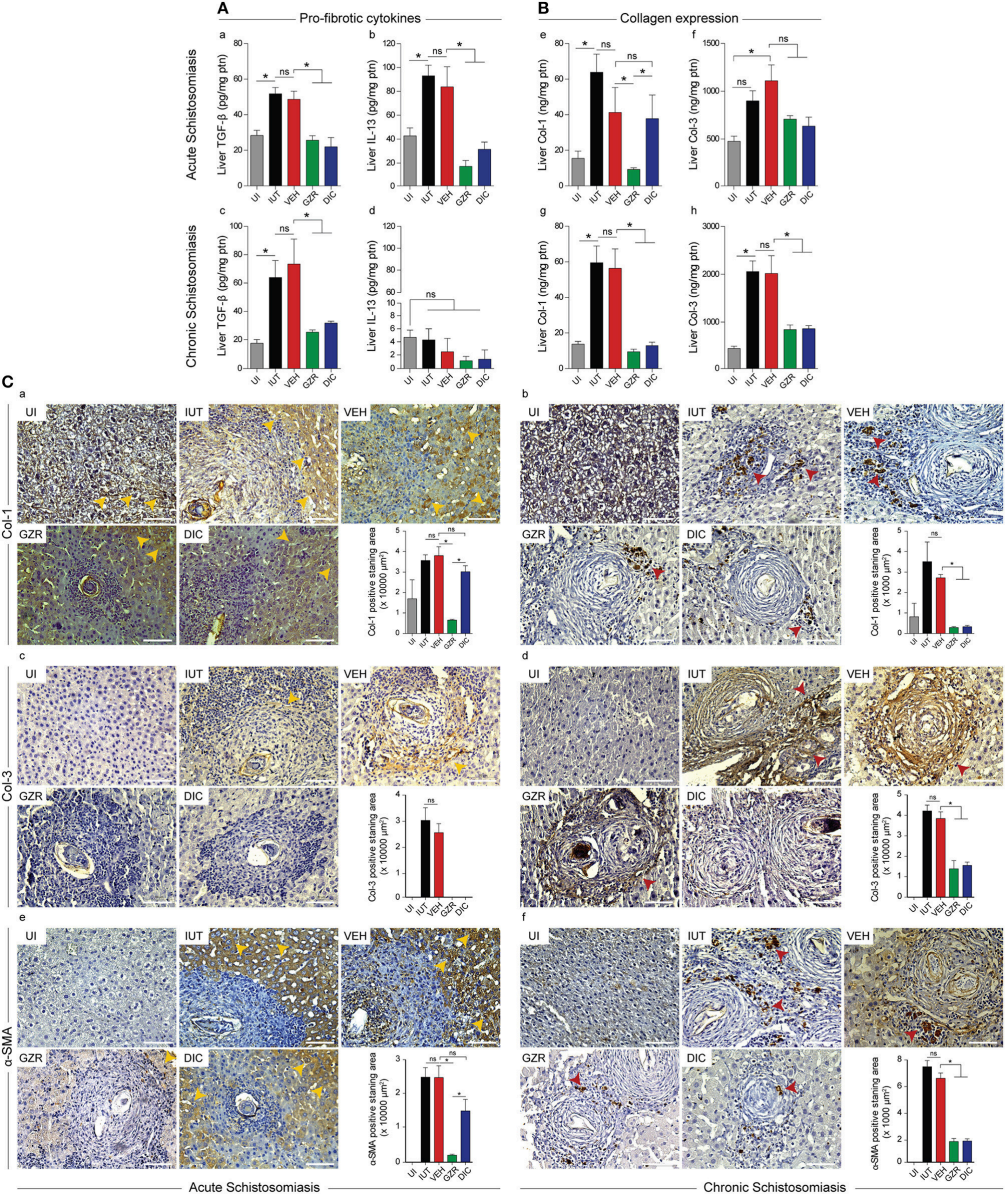
HMGB1 plays a role in egg-induced liver fibrogenesis. **(A,B)** ELISA of liver extracts for TGF-β, IL-13, collagen 1 and 3, in acute and chronic schistosomiasis. The results were presented as the mean ± SEM and were tested by ANOVA Tukey's multiple comparisons test. *P < 0.05 was considered statistically significant; ns—not significant. The *P*-values for all comparisons are available in Table S3. **(C)** Representative photographs of immunohistochemistry (from 5 animals per group) of Col-1, Col-3, and α-SMA in hepatic tissues of animals with acute or chronic schistosomiasis. In acute schistosomiasis, Col-1 and α-SMA were detected intracellularly (b,f, yellow arrows), whereas in chronic schistosomiasis, Col-1 and α-SMA were deposited extracellularly (c,g, red arrows). Col-3 was stained inside the granuloma. Photographs are at a magnification of 40x. Scale bars = 50 μm. The graphs (b–g) represent the quantification of positive staining areas for Col-1, Col-3, and α-SMA in acute and chronic schistosomiasis. Legends of the experimental groups are as depicted in Figure 2.

Since GZR- or DIC-mediated inhibition of HMGB1 culminated in the downregulation of IL-13 (Figure 4G), a cytokine known to be involved in liver fibrosis regulation, we decided to investigate the role of HMGB1 in the pro-fibrotic processes of murine schistosomiasis. Our ELISA showed that the levels of TGF-β and IL-13 were increased in the liver of mice with acute schistosomiasis (Figure 6A, panels a and b). However, mice that were treated with GZR and DIC to inhibit HMGB1 revealed a significant reduction of TGF-β and IL-13 (Figure 6A, panels a and b). In mice with chronic schistosomiasis, inhibition of HMGB1 resulted in lower levels of TGF-β (Figure 6A, panel c). With regard to IL-13, only very low levels of this cytokine were detected in the liver of chronically infected mice, and no significant differences were observed after treatments (Figure 6A, panel d). It is worth noting that the undetectable levels of IL-13 in the sera of mice with chronic schistosomiasis (shown in Figure 4O) could be associated with the lack of IL-13 in the liver extracts of these mice. Because the expression of most of the components of the extracellular matrix depends on TGF-β and IL-13 signaling activation, we investigated the levels of collagen in the liver of acutely or chronically infected mice (Figure 6B). Our data indeed showed that schistosome infections exacerbate the production of collagen 1 (Col-1) and collagen 3 (Col-3), Figure 6B, panels e–h; compare the UT group with the IUT and VEH groups. Because HMGB1 inhibition down-regulated the expression of pro-fibrotic cytokines (Figure 6A), a reduction in collagen levels in the liver of acutely and chronically infected mice was expected. Indeed, our data confirmed that the levels of Col-1 and Col-3 were decreased upon treatments (Figure 6, panels e–h, compare the VEH group with the GZR and DIC groups), except for collagen 1 in mice with acute infections that were treated with DIC (Figure 6B, panel e).

In order to better assess the levels of liver fibrosis, we also used Masson Trichome and Sirius Red staining. With these staining techniques, we confirmed an intense collagen deposition in animals with acute or chronic schistosomiasis that were not treated (IUT) or treated with vehicle (VEH) only (Figure S5). Of note, in the liver of chronically infected mice (lower panels), we could observe extensive areas of fibrosis within several fused isolated granulomas (Figure S5A). Importantly, livers of acutely or chronically infected mice that we treated with HMGB1 inhibitors revealed a significant reduction in collagen deposition, and showing better preserved parenchyma areas (Figure S5A; compare IUT and VEH with GZR and DIC panels of both acute and chronic schistosomiasis). Collagen quantifications are shown in Figure S5B.

We next performed immunohistochemistry of liver slices from infected mice with acute schistosomiasis that were treated with GZR or DIC (Figure 6C). With regard to collagen expression, the livers of mice with acute schistosomiasis in the IUT, VEH, and DIC groups overexpressed Col-1 (Figure 6C, panel a). In the GZR treated-group only, we observed a reduction in the positive staining area for Col-1, which confirmed our ELISA data above (Figure 6B, panel e). Of note, Col-1 was also expressed in uninfected mice, but at reduced levels (Figure 6C, panel a). The immunohistochemistry of chronic schistosomiasis revealed that treatment with GZR or DIC attenuated the extracellular deposition of Col-1 (Figure 6C, panel b, compare the IUT and VEH groups with the GZR and DIC groups).

In regard to Col-3 and another important pro-fibrotic marker, α-smooth muscle actin (α-SMA), our immunohistochemistry revealed high staining of both molecules in the livers of mice with acute and chronic schistosomiasis in the IUT and VEH groups (Figure 6C, panels c and d for Col-3, and Figure 6C, panels e and f for α-SMA), but showed much less reactivity in the livers of mice treated with GZR or DIC (Figure 6C, panels c and d), indicating that HMGB1 modulates key fibrotic factors. The specificity of the immunoreactivity of Col-1, Col-3, and α-SMA was proved by the lack of immunostaining using the secondary anti-rabbit control (Figure S1). All graphics next to the immunohistochemistry panels correspond to the quantification of positive staining areas.

### HMGB1 inhibition ameliorates liver fibrosis and improves the survival of mice with chronic schistosomiasis

**Figure 7:**
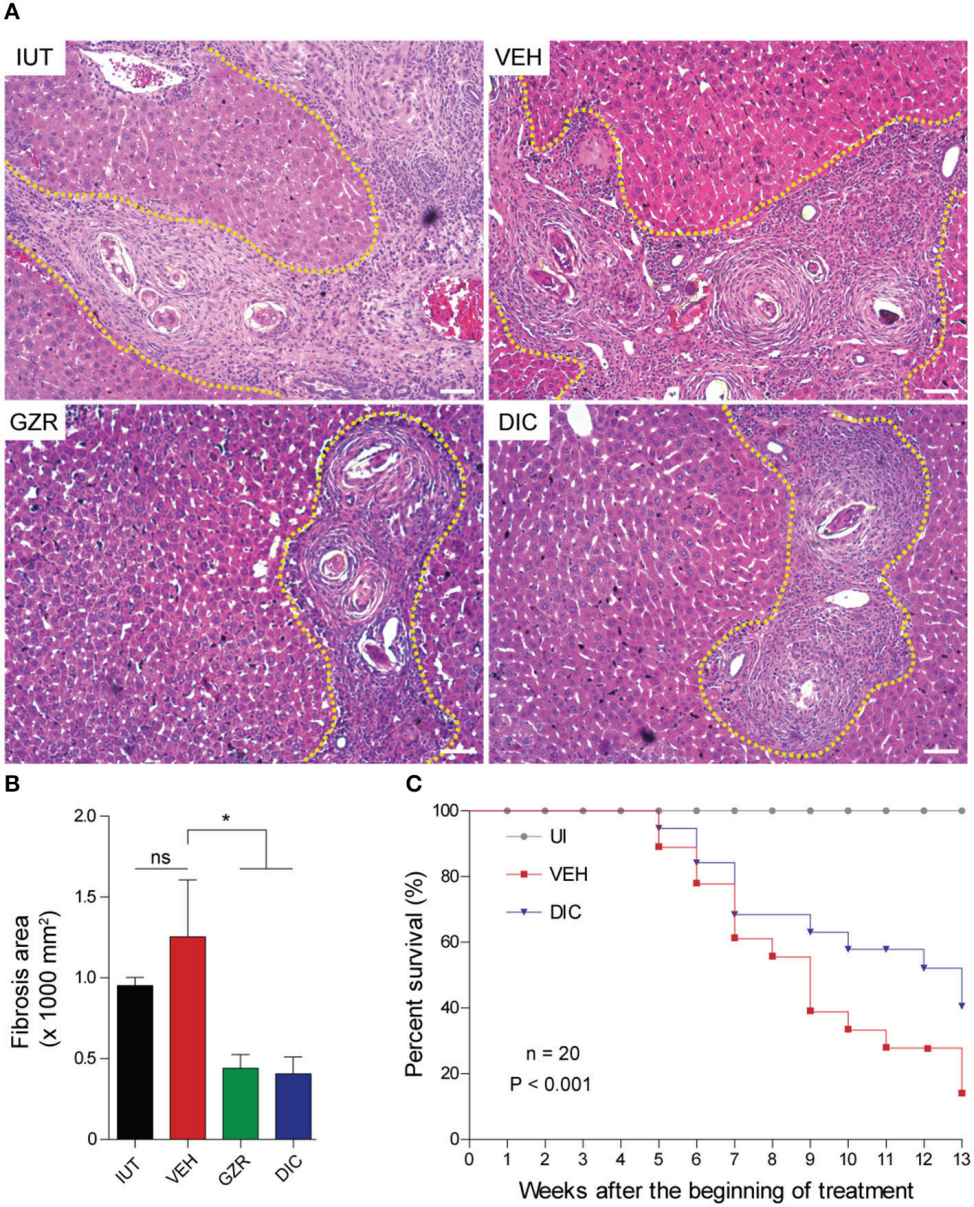
HMGB1 modulates liver fibrosis in chronic schistosomiasis and HMGB1 inhibition improves mouse survival. **(A)** Representative photographs of hematoxylin and eosin-stained sections of livers. Yellow dashed lines depict the areas of liver fibrosis. Photographs are at a magnification of 20x. Scale bars = 20 μm. **(B)** Quantification of the area of liver fibrosis from 15 animals. The measurements were performed using ImageJ software. The results were presented as the mean ± SEM and were tested by ANOVA Tukey's multiple comparisons test. *P < 0.05 was considered statistically significant; ns—not significant. The *P*-values for all comparisons are available in Table S3. **(C)** Survival curve of *S. mansoni-*infected mice (20 per group infected with 80 cercariae) treated with DIC. The survival curve was monitored during the 16 weeks (112 days) of infection. Analysis of the survival curve was performed via a Kaplan-Meier plot, and *P*-values (*P* < 0.0001) were calculated by the log-rank (Mantel-Cox) test. Legends of the experimental groups are as depicted in Figure 2.

We next investigated the role of HMGB1 in the pathogenesis of schistosomiasis. Morphometric analysis of the livers of animals with chronic schistosomiasis revealed extensive fibrosis around the eggs of controlled animals (Figure 7A, IUT, and VEH) whereas in animals that were treated with both HMGB1 inhibitors (GZR or DIC), a healthier hepatic parenchyma with fewer fibrotic areas were observed (Figures 7A,B).

To further confirm the role of HMGB1 in the development of schistosomiasis, we chose to establish a survival curve of infected animals, but we treated the animals only with DIC (and not GZR) due to its ability to inhibit HMGB1 secretion but also because of its novelty *in vivo* experiments [the anti-HMGB1 activity of GZR has been previously tested in animals with different inflammatory diseases (54)]. Thus, animals that were treated with DIC to inhibit HMGB1 pro-inflammatory activity survived longer (40% of survival; blue line with inverted triangle) than animals that received the vehicle alone (10% of survival; red line with squares; Figure 7C).

## Discussion

*Schistosoma mansoni* infection invariably results in liver fibrosis of the host. This fibrosis may be represented by small focal areas of chronic inflammation and excess extracellular matrix deposited in periovular granulomas that are distributed in variable numbers at the periphery of the portal vein system. This condition is noted in 90% of the infected population in endemic areas. Conversely, a minority of infected individuals develops extensive disease with numerous granulomas along the entire extension of the portal space. This latter situation is mainly dependent on special hemodynamic changes created by a heavy worm load with the subsequent production of numerous eggs and represents a severe form of a peculiar chronic hepatopathy. Intestinal schistosomiasis is another well-defined form of chronic schistosomiasis (55) but its relationship with HMGB1 activity was not addressed in this paper.

In this study, we observed high levels of HMGB1 in the sera of mice with acute or chronic schistosomiasis, and the levels were slightly higher in the acute phase. Of note, high levels of HMGB1 were previously detected in the sera of patients suffering from hepatic fibrosis, thus suggesting that HMGB1 is an important and reliable non-invasive marker of the level of fibrosis in patients with hepatitis B (38). In this context, we also observed high levels of HMGB1 in the sera of patients from an endemic area in the State of Minas Gerais, Brazil, who suffer from acute or chronic schistosomiasis.

Based on immunohistochemistry, HMGB1 was predominantly localized in the cytoplasm of hepatocytes surrounding the schistosomotic granuloma in the livers of mice with acute schistosomiasis. However, during chronic infections, HMGB1 was mainly found in the nuclei of hepatocytes. These data suggest that during acute schistosomiasis, hepatocytes were exposed to an intense cellular injury caused by toxins and immunogenic molecules that are released from the eggs (56–58), which culminated in the HMGB1 nuclear-cytoplasm translocation and its subsequent secretion into the sera of infected animals. While the hepatocytes of mice with acute schistosomiasis are certainly the main source of extracellular HMGB1, HMGB1 from the eggs trapped in the liver can also be released (59), and could then, contribute to a lesser extent, to liver fibrosis. Therefore, HMGB1 may play a key role in the regulation of the immune response during the formation of the schistosomotic granuloma.

Previous studies have strongly suggested a role for HMGB1 as a mediator of inflammation in hepatic disorders, and hepatocytes have been considered the main source of extracellular HMGB1 associated with the immune response during acute hepatic failure. In this regard, HMGB1 was localized in the cytoplasm of hepatocytes in models of hepatic fibrosis induced by concanavalin A or carbon tetrachloride (39, 60). Because schistosomiasis is considered an inflammatory hepatic disease and given that hepatocytes are the first liver cells to recognize the presence of *S. mansoni* eggs, we believe that hepatocytes are key players in the formation of the granuloma. Thus, the HMGB1 released by these hepatocytes may act as a major regulatory cytokine in this process by recruiting neutrophils, macrophages, dendritic cells, lymphocytes, and hepatic stellate cells to the areas surrounding the eggs.

To confirm the involvement of HMGB1 in the process of granuloma formation, infected mice were treated with two inhibitors of HMGB1: 1. GZR, a natural triterpene glycoconjugate derived from the root of licorice that physically interacts with HMGB1 and inhibits its chemotactic and mitogenic functions (43); 2. DIC, a five-membered heterocyclic synthetic compound containing a N-O bond that inhibits HMGB1 translocation (42). In the livers of mice under acute infection that received GZR or DIC treatments, HMGB1 was mainly localized in the nuclei of the hepatocytes. These data indicate that both compounds inhibited HMGB1 translocation *in vivo* despite continuous stimulation from the egg toxins and antigens. Importantly, animals treated with GZR or DIC also presented a significant reduction in the area of the granuloma and a decrease in serum cytokine levels. Of note, GZR, or DIC significantly reduced the levels of IL-13, a known pro-fibrotic cytokine involved in the differentiation of hepatic stellate cells into myofibroblasts. Myofibroblasts are key cells in the process of fibrosis that secrete collagen and α-smooth muscle actin (10, 14, 16). In this regard, it is worth pointing out that IL-13 and IFN-γ-double deficient mice showed a greater reduction in fibrosis in animals infected with *S. mansoni* (61). Our data suggest that HMGB1 is secreted by hepatocytes, and that it possibly activates immune cells through TLR4/9 and/or RAGE (28–30), leading to an increase release of IL-13 and other cytokines into the sera of infected mice.

Survival experiments in animals infected with *S. mansoni* revealed a significant role for HMGB1 in mediating pro-inflammatory responses against the eggs, thereby culminating in the process of fibrogenesis. In this context, the hepatic parenchyma of control animals exhibited wide areas of fibrosis, while the inhibition of HMGB1 release resulted in animals with healthier livers and higher survival rates. We believe that this healthier condition of the mice was due to the effect of three main conditions: 1. down-modulation of the two main pro-fibrotic cytokines in the liver: IL-13 and TGF-β; 2. up-regulation of the main anti-inflammatory cytokine IL-10 in the chronic phase. In this context, a protective effect of IL-10 up-regulation has been previously observed in schistosomiasis (62); 3. down-regulation of IL-17A in the chronic phase. Of note, IL-17A plays a major role for full deployment of inflammation and for the development of severe schistosome egg-induced immunopathology (63, 64). To date, efficacious treatment of schistosomiasis relies exclusively on the use of Praziquantel (PZQ) (65), a drug that eliminates adult worms. In clinics, when an individual initiates treatment with PZQ, the liver disease is often previously established due to the presence of eggs. Moreover, despite PZQ treatment, the disease persists due the presence of eggs previously deposited in the liver. These data reinforce the importance of adopting a new approach to treat schistosomiasis that does not exclusively rely on the elimination of adult worms but also interferes with the enhanced immune responses directed against laid eggs. In this respect, the understanding of key determinants of immunomodulation in human schistosomiasis could lead to the development of new drugs to control the disease. Therefore, it would come as no surprise if HMGB1 was elicited as a target. Indeed, our animal survival curves showed an increased survival rate in mice under DIC administration, supporting the potential of HMGB1 inhibitors to treat schistosomiasis. In this regard, it is worth pointing out that several therapeutic agents that target extracellular HMGB1 in several inflammatory diseases are currently in preclinical or clinical development (66).

**Figure 8:**
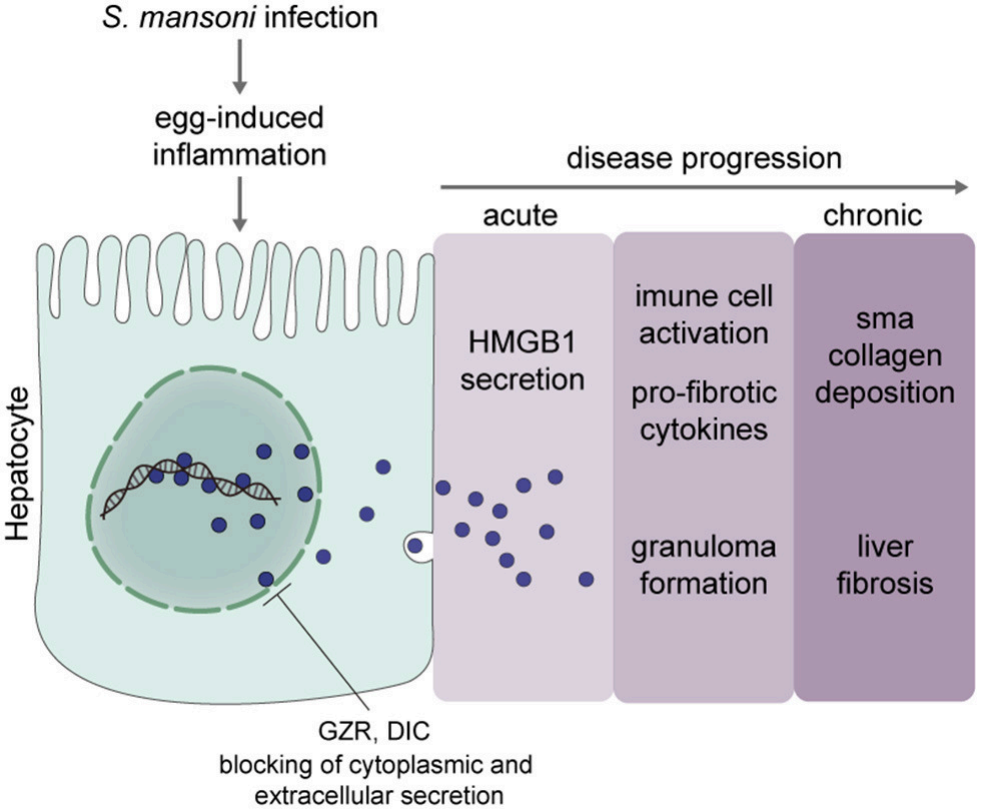
Proposed model of the role of HMGB1 in *S. mansoni* egg-induced liver fibrogenesis. Our hypothesis is that *S. mansoni* egg-induced inflammation triggers the hepatocytes to release HMGB1 from chromatin, likely through acetylation. Modified HMGB1 follows the secretory pathway and in the extracellular milieu, signals for the production of molecules involved in inflammation and components of the extracellular matrix, which ultimately leads to liver fibrosis. HMGB1 seems to play a role in the transition from acute to chronic schistosomiasis. Thus, inhibition of HMGB1 secretion by GZR or DIC improved disease outcomes. It is important to point out that at this stage, we cannot rule out the possibility that GZR or DIC might also interfere with the activity of other alarmins during egg-induced inflammation.

We envision a model (Figure 8) where anti-HMGB1 drugs would lead to an efficacious control of schistosomotic liver fibrosis, as follows: during acute schistosomiasis, secreted egg toxins, and antigens trigger a potent immune reaction and inflammatory response that lead to hepatic tissue damage. HMGB1, which under physiological conditions is an abundant chromosome-bound protein (18), under hepatic damage, can be released from chromatin and secreted to the extracellular milieu. Extracellular HMGB1 will bind to TLR4/9 and/or RAGE (28–30) of the host target cells. The HMGB1-receptor complex will then trigger cell activation to release inflammatory mediators and pro-fibrotic cytokines that lead to cell recruitment to the liver, culminating with the development of granulomatous reaction and fibrosis.

Although we cannot describe at present the exact molecular mechanism by which hepatocyte HMGB1 signals for the pathogenesis of schistosomotic liver fibrosis, the alarmin activity of HMGB1 seems to be a key factor for the typical exacerbation of the immune responses against the eggs.

## Ethics statement

The present study was conducted in accordance with the Declaration of Helsinki (2013) of the World Medical Association and was approved as a written document by the Ethics Committee of UFMG (Protocol ETIC 204/06). Informed written consent was obtained from all participating subjects. Human subjects included children. Parent or guardian of any child participant provided informed consent on their behalf. Sera from patients who suffer from acute or chronic schistosomiasis were provided by Fiocruz—Minas (http://www.cpqrr.fiocruz.br/pg/). Sera from healthy donors were collected in a blood bank of the Laboratório Central Noel Nutels (LACEN—Rio de Janeiro), with their agreement for research use. Blood samples from healthy donors were negative for HIV and hepatitis. This study was performed in strict accordance with the recommendations in the Guide for the Care and Use of Laboratory Animals. The protocol was approved as a written document provided by the Committee for the Evaluation of Animal Use for Research from the Federal University of Rio de Janeiro, CEUA-UFRJ (Permit Number: IBQM079-05/16). All efforts were made to minimize animal suffering.

## Author contributions

AV performed experiments, collected and analyzed data, and wrote the manuscript. VC, DA, RG, HdS, FX, AP, JG, MCF, TP, and LL performed experiments, collected, and analyzed data. CB, RdO, and JL provided resources. MRF was responsible for supervision, funding, and writing.

### Conflict of interest statement

The authors declare that the research was conducted in the absence of any commercial or financial relationships that could be construed as a potential conflict of interest.

## Supplementary material

Figure S1 Negative control of the immunohistochemistry analyses. Liver slices from control and infected mice that were incubated with secondary anti-rabbit antibodies exhibited no immunostaining. Photomicrographs are presented at 40x magnification. Scale bars = 50 μm.

Figure S2 Egg burden in animals with acute or chronic schistosomiasis. No differences were identified among the groups. The results were presented as the mean ± SEM and were tested by ANOVA Tukey's multiple comparisons test.

Figure S3 *S. mansoni* adult worm viability after DIC treatment. Adult worms were recovered from mice via perfusion of the mesenteric veins (67) after 6 weeks of infection. Five pairs of worms were cultured in the presence or absence of vehicle (propylene-glycol 0.025%) or DIC (150 μM) for 72 h. Worm viability was measured by adenosine triphosphate (ATP) dosage, as previously described (68), using CellTiter-Glo®reagent (Promega, Wisconsin, USA). Three independent experiments were performed. The results were presented as the mean ± SEM and were tested by ANOVA Tukey's multiple comparisons test. No differences were identified among the groups.

Figure S4 Primary hepatocytes release HMGB1 in the presence of SEA. Mice were perfused through the portal vein with HANKS A and then HANKS B medium containing 0.05% collagenase (Roche Applied Science, Indianapolis, IN) and the perfused liver tissue was passed through a 40-μm nylon mesh filter (69, 70). Primary hepatocytes were cultured at 37°C in 5% CO_2_/95% O_2_ in Williams' medium E (Sigma-Aldrich, Missouri, USA) containing 10% fetal bovine serum (Gibco), 50 units/mL penicillin, 50 g/mL streptomycin (Sigma-Aldrich, Missouri, USA) were plated on collagen-coated coverslips (50 μg/mL) (BD Biosciences, San Jose, CA). Primary hepatocytes were treated with DIC or GZR at 200 μM and were challenged with 10 μg/mL of soluble-egg antigens (SEA) for 24 h. **(A)** Intracellular HMGB1 was visualized with green immunofluorescence FITC-staining. Nuclei were stained with DAPI. BF: bright field. Scale bar 50 μm. **(B)** Negative control of the above experiment. **(C)** Detection of HMGB1 in the supernatant of the hepatocyte's culture by Western blot. Membrane stained with Ponceau S was used as our loading control. **(D)** The cell viability was measured by MTT assay.

Figure S5 HMGB1 inhibition reduces collagen deposition in the liver of mice with schistosomiasis. **(A)** Masson Trichome and Sirius Red staining were used to determine the collagen content and distribution. For Masson staining the collagen fibers are represented by the cyan color and the cell nuclei by purple. For Sirius red staining, the red color represents the collagen fibers. Photographs are at a magnification of 10x. Scale bars = 50 μm. **(B)** Quantifications of the collagen content in liver slices. The results were presented as the mean ± SEM and were tested by ANOVA Tukey's multiple comparisons test. *P* < 0.05 was considered statistically significant; ns—not significant.

Table S1 Demographic data of patients with schistosomiasis. Sera from schistosomotic patients were kindly donated by Instituto René Rachou—Fiocruz Minas. Sera from healthy donors were collected in a blood bank of the Laboratório Central Noel Nutels (LACEN - Rio de Janeiro), with their agreement for research use. Blood from healthy donors were tested and were negative for HIV and hepatitis.

Table S2 Effect of DIC treatment on body weight, relative organs weights, ALT, AST and creatinine in healthy mice. At the end of 56 and 112 days post-treatment, healthy untreated (UT) mice and healthy mice with treated with DIC (DIC) were weighed before sacrifice. Relative liver and spleen weights were calculated as following: Relative organ weight = (absolute weight / body weight) × 100. No significant differences (*p* > 0.05) were observed with relative liver and spleen weights between the UI and DIC groups. Activities of alanine aminotransferase (ALT), aspartate aminotransferase (AST) and creatinine levels were measured in the sera of UT and DIC healthy mice at days 56 and 112 post-treatment. ^a^ ALT values were only significantly different (*p* < 0.05) between UT and DIC groups at day 112 post-treatment. AST and creatinine values did not show significant differences in both treatments. Data are expressed as the mean ± SD of 20 mice per group. The results were tested by Student's *t*-test.

Table S3 *P*-values of the statistical analyses of all data presented in this work using one-way ANOVA test.

## References

[1] Symmers D. Pathogenesis of liver cirrhosis in schistosomiasis. J Am Med Assoc. (1951) 147:304–5. 10.1001/jama.1951.03670210016005 14873476

[2] Manzella A Ohtomo K Monzawa S Lim JH. Schistosomiasis of the liver. Abdom Imaging (2008) 33:144–50. 10.1007/s00261-007-9329-7 17912583

[3] Gryseels B Polman K Clerinx J Kestens L. Human schistosomiasis. Lancet (2006) 368:1106–18. 10.1016/S0140-6736(06)69440-3 16997665

[4] Ross AG Bartley PB Sleigh AC Olds GR Li Y Williams GM. Schistosomiasis. N Engl J Med. (2002) 346:1212–20. 10.1056/NEJMra012396 11961151

[5] Grimes JE Templeton MR. Geostatistical modelling of schistosomiasis prevalence. Lancet Infect Dis. (2015) 15:869–70. 10.1016/S1473-3099(15)00067-5 26004858

[6] Gressner OA Weiskirchen R Gressner AM. Evolving concepts of liver fibrogenesis provide new diagnostic and therapeutic options. Comp Hepatol. (2007) 6:7. 10.1186/1476-5926-6-7 17663771 PMC1994681

[7] Boros DL. Immunopathology of Schistosoma mansoni infection. Clin Microbiol Rev. (1989) 2:250–69. 10.1128/CMR.2.3.250 2504481 PMC358119

[8] Chuah C Jones MK Burke ML McManus DP Gobert GN. Cellular and chemokine-mediated regulation in schistosome-induced hepatic pathology. Trends Parasitol. (2014) 30:141–50. 10.1016/j.pt.2013.12.009 24433721

[9] Wynn TA Thompson RW Cheever AW Mentink-Kane MM. Immunopathogenesis of schistosomiasis. Immunol Rev. (2004) 201:156–67. 10.1111/j.0105-2896.2004.00176.x 15361239

[10] Friedman SL. Mechanisms of hepatic fibrogenesis. Gastroenterology (2008) 134:1655–69. 10.1053/j.gastro.2008.03.003 18471545 PMC2888539

[11] Iredale JP. Models of liver fibrosis: exploring the dynamic nature of inflammation and repair in a solid organ. J Clin Invest. (2007) 117:539–48. 10.1172/JCI30542 17332881 PMC1804370

[12] Lee UE Friedman SL. Mechanisms of hepatic fibrogenesis. Best Pract Res Clin Gastroenterol. (2011) 25:195–206. 10.1016/j.bpg.2011.02.005 21497738 PMC3079877

[13] Liu Y Meyer C Muller A Herweck F Li Q Mullenbach R. IL-13 induces connective tissue growth factor in rat hepatic stellate cells via TGF-beta-independent Smad signaling. J Immunol. (2011) 187:2814–23. 10.4049/jimmunol.1003260 21804025

[14] Mann DA Marra F. Fibrogenic signalling in hepatic stellate cells. J Hepatol. (2010) 52:949–50. 10.1016/j.jhep.2010.02.005 20395006

[15] Bataller R Brenner DA. Hepatic stellate cells as a target for the treatment of liver fibrosis. Semin Liver Dis. (2001) 21:437–51. 10.1055/s-2001-17558 11586471

[16] Gressner AM Chunfang G. A cascade-mechanism of fat storing cell activation forms the basis of the fibrogenic reaction of the liver. Verh Dtsch Ges Pathol. (1995) 79:1–14. 8600672

[17] Gerlitz G Hock R Ueda T Bustin M. The dynamics of HMG protein-chromatin interactions in living cells. Biochem Cell Biol. (2009) 87:127–37. 10.1139/O08-110 19234529 PMC3459335

[18] Goodwin GH Sanders C Johns EW. A new group of chromatin-associated proteins with a high content of acidic and basic amino acids. Eur J Biochem. (1973) 38:14–9. 10.1111/j.1432-1033.1973.tb03026.x 4774120

[19] Travers AA. Priming the nucleosome: a role for HMGB proteins? EMBO Rep. (2003) 4:131–6. 10.1038/sj.embor.embor741 12612600 PMC1315838

[20] Andersson U Erlandsson-Harris H Yang H Tracey KJ. HMGB1 as a DNA-binding cytokine. J Leukoc Biol. (2002) 72:1084–91. 10.1189/jlb.72.6.1084 12488489

[21] Scaffidi P Misteli T Bianchi ME. Release of chromatin protein HMGB1 by necrotic cells triggers inflammation. Nature (2002) 418:191–5. 10.1038/nature00858 12110890

[22] Bell CW Jiang W Reich CF 3rd Pisetsky DS. The extracellular release of HMGB1 during apoptotic cell death. Am J Physiol Cell Physiol. (2006) 291:C1318–25. 10.1152/ajpcell.00616.2005 16855214

[23] Diener KR Al-Dasooqi N Lousberg EL Hayball JD. The multifunctional alarmin HMGB1 with roles in the pathophysiology of sepsis and cancer. Immunol Cell Biol. (2013) 91:443–50. 10.1038/icb.2013.25 23797067

[24] Li J Wang H Mason JM Levine J Yu M Ulloa L. Recombinant HMGB1 with cytokine-stimulating activity. J Immunol Methods (2004) 289:211–23. 10.1016/j.jim.2004.04.019 15251426

[25] Pisetsky DS Erlandsson-Harris H Andersson U. High-mobility group box protein 1 (HMGB1): an alarmin mediating the pathogenesis of rheumatic disease. Arthritis Res Ther. (2008) 10:209. 10.1186/ar2440 18598385 PMC2483460

[26] Wang H Bloom O Zhang M Vishnubhakat JM Ombrellino M Che J. HMG-1 as a late mediator of endotoxin lethality in mice. Science (1999) 285:248–51. 10.1126/science.285.5425.248 10398600

[27] Wang H Yang H Czura CJ Sama AE Tracey KJ. HMGB1 as a late mediator of lethal systemic inflammation. Am J Respir Crit Care Med. (2001) 164(10 Pt 1):1768–73. 10.1164/ajrccm.164.10.2106117 11734424

[28] Ivanov S Dragoi AM Wang X Dallacosta C Louten J Musco G. A novel role for HMGB1 in TLR9-mediated inflammatory responses to CpG-DNA. Blood (2007) 110:1970–81. 10.1182/blood-2006-09-044776 17548579 PMC1976374

[29] Park JS Svetkauskaite D He Q Kim JY Strassheim D Ishizaka A. Involvement of toll-like receptors 2 and 4 in cellular activation by high mobility group box 1 protein. J Biol Chem. (2004) 279:7370–7. 10.1074/jbc.M306793200 14660645

[30] Hori O Brett J Slattery T Cao R Zhang J Chen JX. The receptor for advanced glycation end products (RAGE) is a cellular binding site for amphoterin. Mediation of neurite outgrowth and co-expression of rage and amphoterin in the developing nervous system. J Biol Chem. (1995) 270:25752–61. 10.1074/jbc.270.43.25752 7592757

[31] Andersson U Wang H Palmblad K Aveberger AC Bloom O Erlandsson-Harris H. High mobility group 1 protein (HMG-1) stimulates proinflammatory cytokine synthesis in human monocytes. J Exp Med. (2000) 192:565–70. 10.1084/jem.192.4.565 10952726 PMC2193240

[32] Degryse B Bonaldi T Scaffidi P Muller S Resnati M Sanvito F. The high mobility group (HMG) boxes of the nuclear protein HMG1 induce chemotaxis and cytoskeleton reorganization in rat smooth muscle cells. J Cell Biol. (2001) 152:1197–206. 10.1083/jcb.152.6.1197 11257120 PMC2199202

[33] El Gazzar M. HMGB1 modulates inflammatory responses in LPS-activated macrophages. Inflamm Res. (2007) 56:162–7. 10.1007/s00011-006-6112-0 17522814

[34] Fages C Nolo R Huttunen HJ Eskelinen E Rauvala H. Regulation of cell migration by amphoterin. J Cell Sci. (2000) 113 (Pt 4):611–20. 10652254 10.1242/jcs.113.4.611

[35] Li W Xu Q Deng Y Yang Z Xing S Zhao X. High-mobility group box 1 accelerates lipopolysaccharide-induced lung fibroblast proliferation *in vitro*: involvement of the NF-kappaB signaling pathway. Lab Invest. (2015) 95:635–47. 10.1038/labinvest.2015.44 25867768

[36] Li LC Gao J Li J. Emerging role of HMGB1 in fibrotic diseases. J Cell Mol Med. (2014) 18:2331–9. 10.1111/jcmm.12419 25284457 PMC4302638

[37] Kao YH Jawan B Goto S Hung CT Lin YC Nakano T. High-mobility group box 1 protein activates hepatic stellate cells *in vitro*. Transplant Proc. (2008) 40:2704–5. 10.1016/j.transproceed.2008.07.055 18929840

[38] Albayrak A Uyanik MH Cerrah S Altas S Dursun H Demir M. Is HMGB1 a new indirect marker for revealing fibrosis in chronic hepatitis and a new therapeutic target in treatment? Viral Immunol. (2010) 23:633–8. 10.1089/vim.2010.0080 21142449

[39] Tu CT Yao QY Xu BL Wang JY Zhou CH Zhang SC. Protective effects of curcumin against hepatic fibrosis induced by carbon tetrachloride: modulation of high-mobility group box 1, Toll-like receptor 4 and 2 expression. Food Chem Toxicol. (2012) 50:3343–51. 10.1016/j.fct.2012.05.050 22683883

[40] Ghidini E Capelli AM Carnini C Cenacchi V Marchini G Virdis A. Discovery of a novel isoxazoline derivative of prednisolone endowed with a robust anti-inflammatory profile and suitable for topical pulmonary administration. Steroids (2015) 95:88–95. 10.1016/j.steroids.2014.12.016 25556984

[41] Stojanovic I Cuzzocrea S Mangano K Mazzon E Miljkovic D Wang M. *In vitro, ex vivo* and *in vivo* immunopharmacological activities of the isoxazoline compound VGX-1027: modulation of cytokine synthesis and prevention of both organ-specific and systemic autoimmune diseases in murine models. Clin Immunol. (2007) 123:311–23. 10.1016/j.clim.2007.03.004 17449326

[42] Vicentino AR Carneiro VC Amarante Ade M Benjamim CF de Aguiar AP Fantappie MR. Evaluation of 3-(3-chloro-phenyl)-5-(4-pyridyl)-4,5-dihydroisoxazole as a novel anti-inflammatory drug candidate. PLoS ONE (2012) 7:e39104. 10.1371/journal.pone.0039104 22723938 PMC3377599

[43] Mollica L De Marchis F Spitaleri A Dallacosta C Pennacchini D Zamai M. Glycyrrhizin binds to high-mobility group box 1 protein and inhibits its cytokine activities. Chem Biol. (2007) 14:431–41. 10.1016/j.chembiol.2007.03.007 17462578

[44] Kiso Y Tohkin M Hikino H Hattori M Sakamoto T Namba T. Mechanism of antihepatotoxic activity of glycyrrhizin. I: Effect on free radical generation and lipid peroxidation. Planta Med. (1984) 50:298–302. 10.1055/s-2007-969714 6505079

[45] Cheever AW. Conditions affecting the accuracy of potassium hydroxide digestion techniques for counting Schistosoma mansoni eggs in tissues. Bull World Health Organ. (1968) 39:328–31. 4881073 PMC2554554

[46] Allonso D Belgrano FS Calzada N Guzman MG Vazquez S Mohana-Borges R. Elevated serum levels of high mobility group box 1 (HMGB1) protein in dengue-infected patients are associated with disease symptoms and secondary infection. J Clin Virol. (2012) 55:214–9. 10.1016/j.jcv.2012.07.010 22884669

[47] Belgrano FS de Abreu da Silva IC Bastos de Oliveira FM Fantappie MR Mohana-Borges R. Role of the acidic tail of high mobility group protein B1 (HMGB1) in protein stability and DNA bending. PLoS ONE (2013) 8:e79572. 10.1371/journal.pone.0079572 24255708 PMC3821859

[48] Oktayoglu P Em S Tahtasiz M Bozkurt M Ucar D Yazmalar L. Elevated serum levels of high mobility group box protein 1 (HMGB1) in patients with ankylosing spondylitis and its association with disease activity and quality of life. Rheumatol Int. (2013) 33:1327–31. 10.1007/s00296-012-2578-y 23143556

[49] Schierbeck H Pullerits R Pruunsild C Fischer M Holzinger D Laestadius A. HMGB1 levels are increased in patients with juvenile idiopathic arthritis, correlate with early onset of disease, and are independent of disease duration. J Rheumatol. (2013) 40:1604–13. 10.3899/jrheum.120987 23858044

[50] Harris HE Andersson U Pisetsky DS. HMGB1: a multifunctional alarmin driving autoimmune and inflammatory disease. Nat Rev Rheumatol. (2012) 8:195–202. 10.1038/nrrheum.2011.222 22293756

[51] Hoffmann KF Cheever AW Wynn TA. IL-10 and the dangers of immune polarization: excessive type 1 and type 2 cytokine responses induce distinct forms of lethal immunopathology in murine schistosomiasis. J Immunol. (2000) 164:6406–16. 10.4049/jimmunol.164.12.6406 10843696

[52] Magalhaes A Miranda DG Miranda RG Araujo MI Jesus AA Silva A. Cytokine profile associated with human chronic schistosomiasis mansoni. Mem Inst Oswaldo Cruz. (2004) 99(5 Suppl. 1):21–6. 10.1590/S0074-02762004000900004 15486630

[53] Morais CN Souza JR Melo WG Aroucha ML Miranda P Domingues AL. Cytokine profile associated with chronic and acute human schistosomiasis mansoni. Mem Inst Oswaldo Cruz. (2008) 103:561–8. 10.1590/S0074-02762008000600009 18949326

[54] Xiang K Cheng L Luo Z Ren J Tian F Tang L. Glycyrrhizin suppresses the expressions of HMGB1 and relieves the severity of traumatic pancreatitis in rats. PLoS ONE (2014) 9:e115982. 10.1371/journal.pone.0115982 25541713 PMC4277455

[55] Elbaz T Esmat G. Hepatic and intestinal schistosomiasis: review. J Adv Res. (2013) 4:445–52. 10.1016/j.jare.2012.12.001 25685451 PMC4293886

[56] Abdulla MH Lim KC McKerrow JH Caffrey CR. Proteomic identification of IPSE/alpha-1 as a major hepatotoxin secreted by Schistosoma mansoni eggs. PLoS Negl Trop Dis. (2011) 5:e1368. 10.1371/journal.pntd.0001368 22039561 PMC3201919

[57] Everts B Perona-Wright G Smits HH Hokke CH van der Ham AJ Fitzsimmons CM. Omega-1, a glycoprotein secreted by Schistosoma mansoni eggs, drives Th2 responses. J Exp Med. (2009) 206:1673–80. 10.1084/jem.20082460 19635864 PMC2722183

[58] Fitzsimmons CM Schramm G Jones FM Chalmers IW Hoffmann KF Grevelding CG. Molecular characterization of omega-1: a hepatotoxic ribonuclease from Schistosoma mansoni eggs. Mol Biochem Parasitol. (2005) 144:123–7. 10.1016/j.molbiopara.2005.08.003 16143411

[59] de Abreu da Silva IC Carneiro VC Maciel Rde M da Costa RF Furtado DR de Oliveira FM CK2 phosphorylation of Schistosoma mansoni HMGB1 protein regulates its cellular traffic and secretion but not its DNA transactions. PLoS ONE (2011) 6:e23572 10.1371/journal.pone.0023572 21887276 PMC3160966

[60] Gong Q Zhang H Li JH Duan LH Zhong S Kong XL. High-mobility group box 1 exacerbates concanavalin A-induced hepatic injury in mice. J Mol Med. (2010) 88:1289–98. 10.1007/s00109-010-0681-7 20848269

[61] Ramalingam TR Gieseck RL Acciani TH Hart KM Cheever AW Mentink-Kane MM. Enhanced protection from fibrosis and inflammation in the combined absence of IL-13 and IFN-gamma. J Pathol. (2016) 239:344–54. 10.1002/path.4733 27125685 PMC4915976

[62] Hesse M Piccirillo CA Belkaid Y Prufer J Mentink-Kane M Leusink M. The pathogenesis of schistosomiasis is controlled by cooperating IL-10-producing innate effector and regulatory T cells. J Immunol. (2004) 172:3157–66. 10.4049/jimmunol.172.5.3157 14978122

[63] Rutitzky LI Lopes da Rosa JR Stadecker MJ. Severe CD4 T cell-mediated immunopathology in murine schistosomiasis is dependent on IL-12p40 and correlates with high levels of IL-17. J Immunol. (2005) 175:3920–6. 10.4049/jimmunol.175.6.3920 16148138

[64] Rutitzky LI Stadecker MJ. Exacerbated egg-induced immunopathology in murine Schistosoma mansoni infection is primarily mediated by IL-17 and restrained by IFN-gamma. Eur J Immunol. (2011) 41:2677–87. 10.1002/eji.201041327 21660933 PMC3679923

[65] Zhou YB Zhao GM Jiang QW. Effects of the praziquantel-based control of schistosomiasis japonica in China. Ann Trop Med Parasitol. (2007) 101:695–703. 10.1179/136485907X241488 18028731

[66] Musumeci D Roviello GN Montesarchio D. An overview on HMGB1 inhibitors as potential therapeutic agents in HMGB1-related pathologies. Pharmacol Ther. (2014) 141:347–57. 10.1016/j.pharmthera.2013.11.001 24220159

[67] Carneiro VC de Abreu da Silva IC Torres EJ Caby S Lancelot J Vanderstraete M. Epigenetic changes modulate schistosome egg formation and are a novel target for reducing transmission of schistosomiasis. PLoS Pathogens (2014) 10:e1004116. 10.1371/journal.ppat.1004116 24809504 PMC4014452

[68] Lalli C Guidi A Gennari N Altamura S Bresciani A Ruberti G. Development and validation of a luminescence-based, medium-throughput assay for drug screening in Schistosoma mansoni. PLoS Negl Trop Dis. (2015) 9:e0003484. 10.1371/journal.pntd.0003484 25635836 PMC4312041

[69] Fonseca MC Franca A Florentino RM Fonseca RC Melo AC Vidigal PTV Cholesterol-enriched membrane microdomains are needed for insulin signaling and proliferation in hepatic cells. Am J Physiol Gastrointest Liver Physiol. (2018) 15:G80–94. 10.1152/ajpgi.00008.2018 PMC6109708 29471671

[70] Cruz LN Guerra MT Kruglov E Mennone A Garcia CR Chen J. Regulation of multidrug resistance-associated protein 2 by calcium signaling in mouse liver. Hepatology (2010) 52:327–37. 10.1002/hep.23625 20578149 PMC3025771

