# Supplementary figures and images for: Emerging Role of HMGB1 in the Pathogenesis of Schistosomiasis Liver Fibrosis
Source: Front Immunol. 2018 Sep 12;9:1979. doi: 10.3389/fimmu.2018.01979 (PMC6143665; doi:10.3389/fimmu.2018.01979)

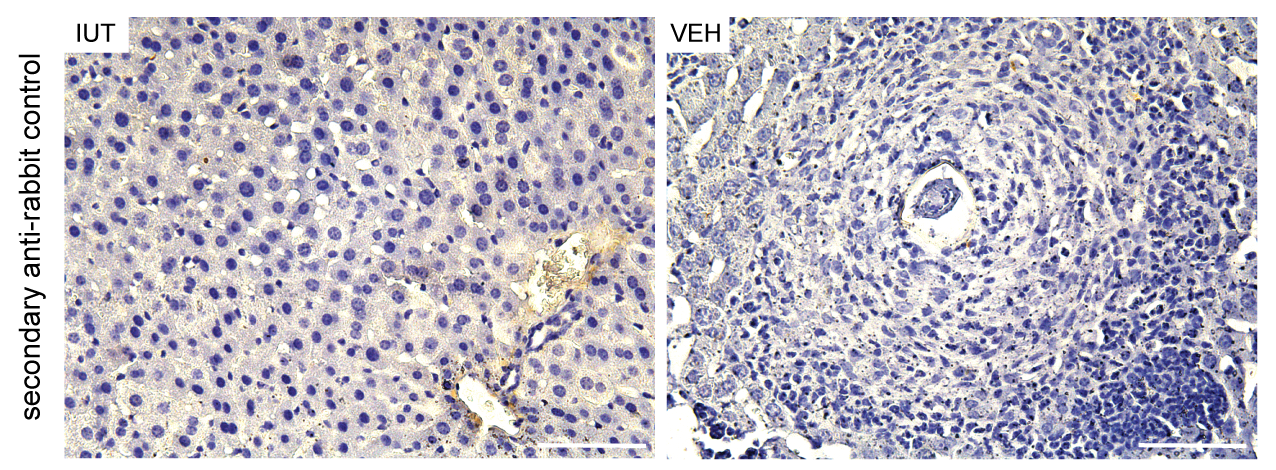

Supplement: Figure S1 — Negative control of the immunohistochemistry analyses. Liver slices from control and infected mice that were incubated with secondary anti-rabbit antibodies exhibited no immunostaining. Photomicrographs are presented at 40x magnification. Scale bars = 50 μm. [file Image_1.TIF]

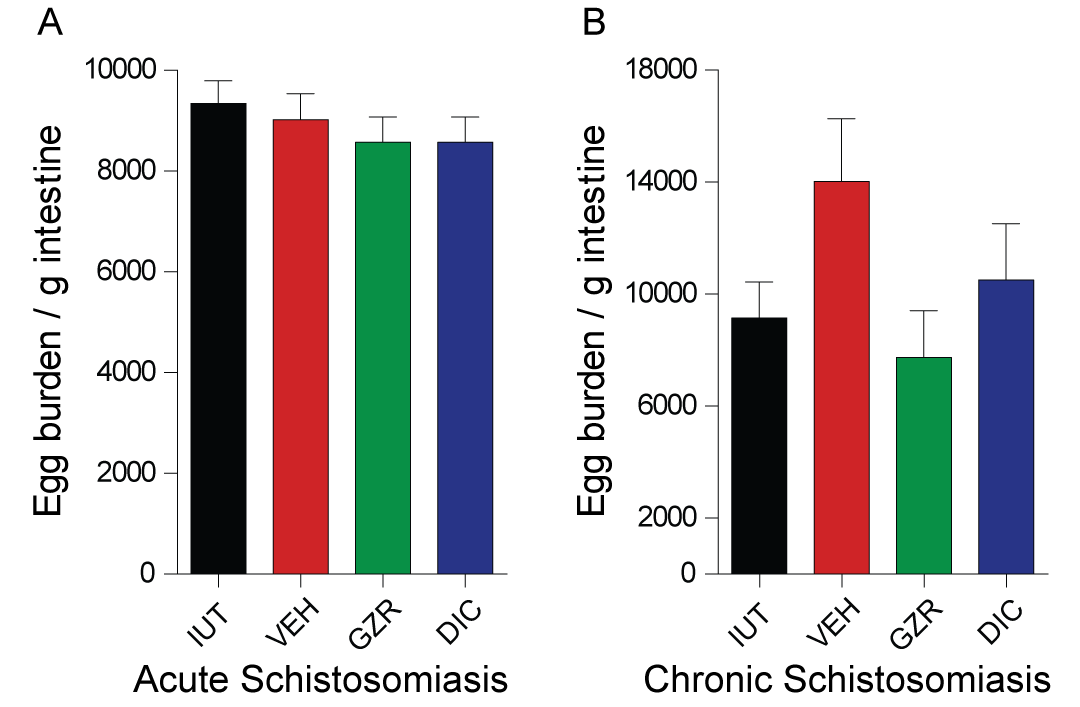

Supplement: Figure S2 — Egg burden in animals with acute or chronic schistosomiasis. No differences were identified among the groups. The results were presented as the mean ± SEM and were tested by ANOVA Tukey's multiple comparisons test. [file Image_2.TIF]

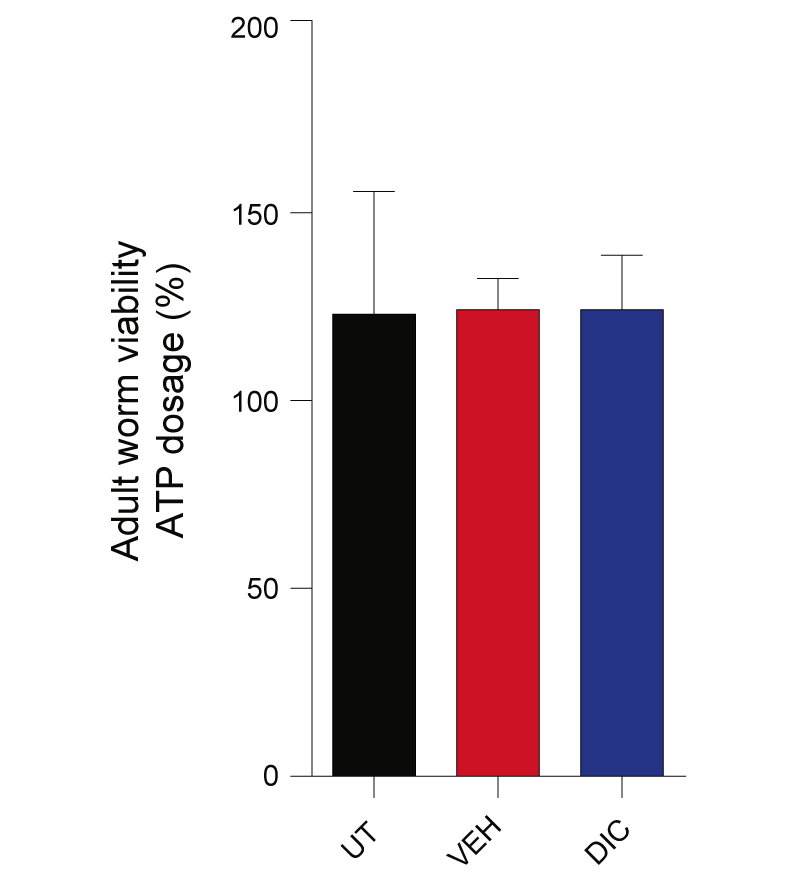

Supplement: Figure S3 — S. mansoni adult worm viability after DIC treatment. Adult worms were recovered from mice via perfusion of the mesenteric veins (67) after 6 weeks of infection. Five pairs of worms were cultured in the presence or absence of vehicle (propylene-glycol 0.025%) or DIC (150 μM) for 72 h. Worm viability was measured by adenosine triphosphate (ATP) dosage, as previously described (68), using CellTiter-Glo®reagent (Promega, Wisconsin, USA). Three independent experiments were performed. The results were presented as the mean ± SEM and were tested by ANOVA Tukey's multiple comparisons test. No differences were identified among the groups. [file Image_3.TIF]

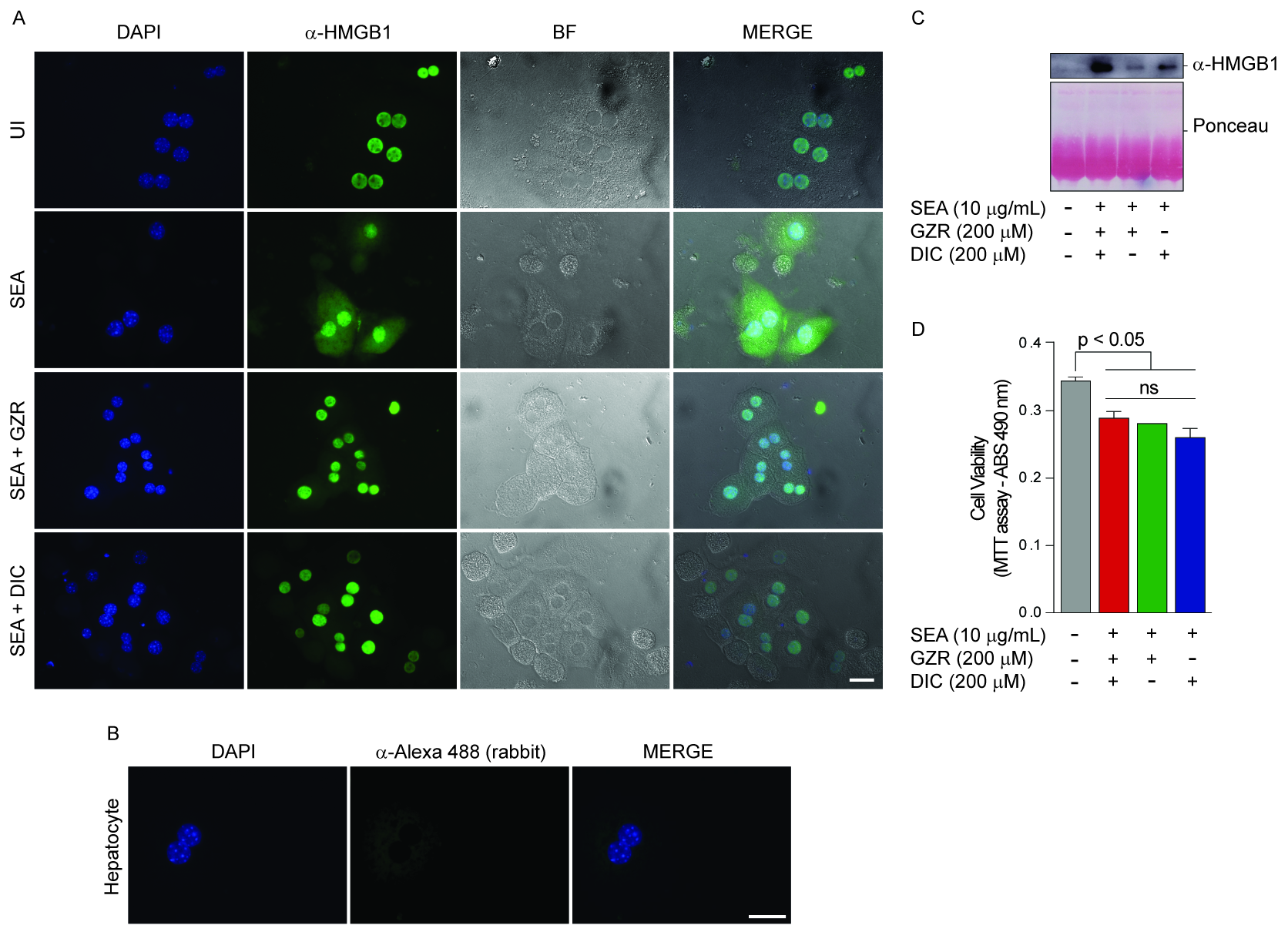

Supplement: Figure S4 — Primary hepatocytes release HMGB1 in the presence of SEA. Mice were perfused through the portal vein with HANKS A and then HANKS B medium containing 0.05% collagenase (Roche Applied Science, Indianapolis, IN) and the perfused liver tissue was passed through a 40-μm nylon mesh filter (69, 70). Primary hepatocytes were cultured at 37°C in 5% CO2/95% O2 in Williams' medium E (Sigma-Aldrich, Missouri, USA) containing 10% fetal bovine serum (Gibco), 50 units/mL penicillin, 50 g/mL streptomycin (Sigma-Aldrich, Missouri, USA) were plated on collagen-coated coverslips (50 μg/mL) (BD Biosciences, San Jose, CA). Primary hepatocytes were treated with DIC or GZR at 200 μM and were challenged with 10 μg/mL of soluble-egg antigens (SEA) for 24 h. (A) Intracellular HMGB1 was visualized with green immunofluorescence FITC-staining. Nuclei were stained with DAPI. BF: bright field. Scale bar 50 μm. (B) Negative control of the above experiment. (C) Detection of HMGB1 in the supernatant of the hepatocyte's culture by Western blot. Membrane stained with Ponceau S was used as our loading control. (D) The cell viability was measured by MTT assay. [file Image_4.TIF]

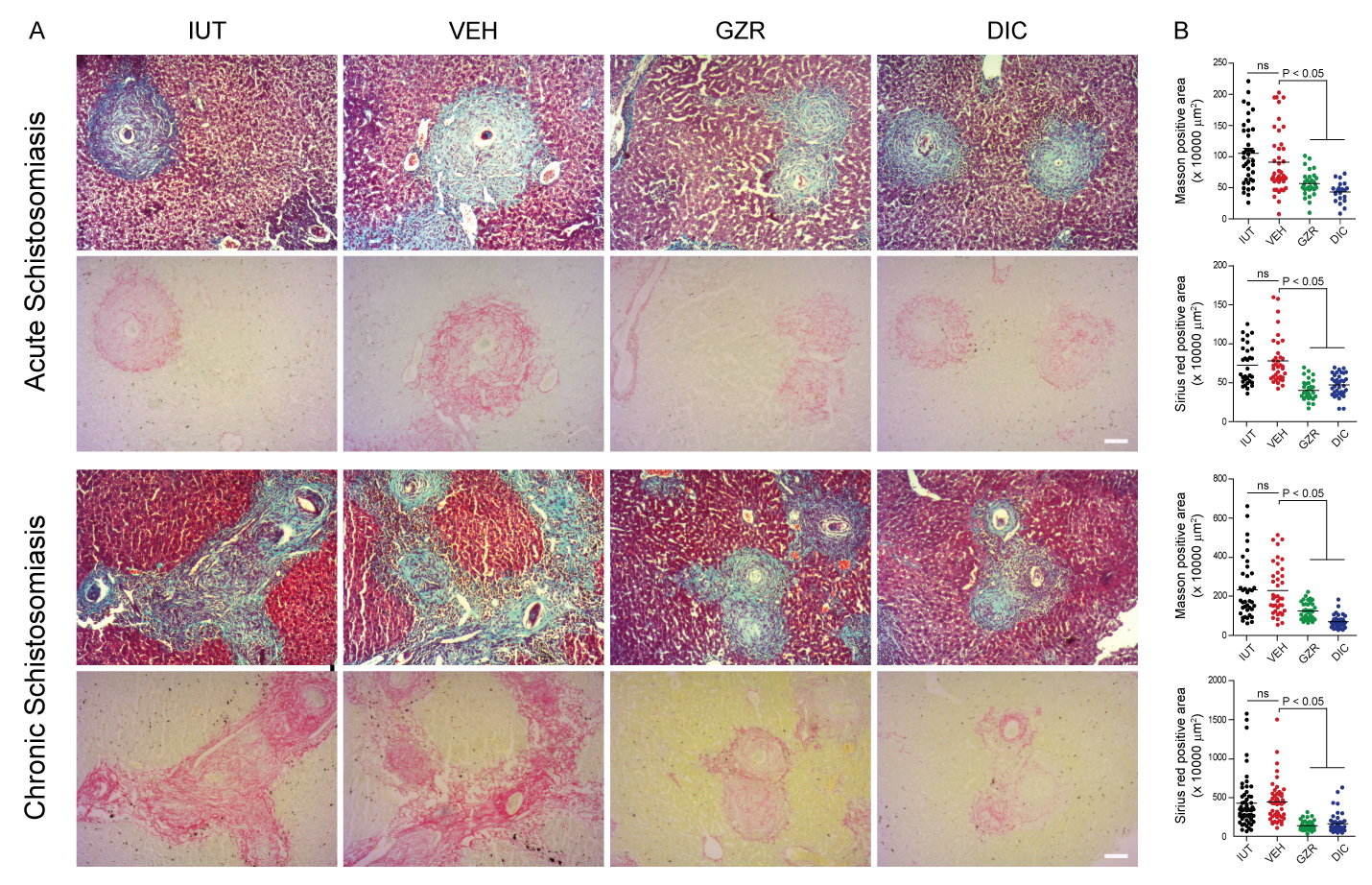

Supplement: Figure S5 — HMGB1 inhibition reduces collagen deposition in the liver of mice with schistosomiasis. (A) Masson Trichome and Sirius Red staining were used to determine the collagen content and distribution. For Masson staining the collagen fibers are represented by the cyan color and the cell nuclei by purple. For Sirius red staining, the red color represents the collagen fibers. Photographs are at a magnification of 10x. Scale bars = 50 μm. (B) Quantifications of the collagen content in liver slices. The results were presented as the mean ± SEM and were tested by ANOVA Tukey's multiple comparisons test. P < 0.05 was considered statistically significant; ns—not significant. [file Image_5.TIF]
