# Supplementary material for: Emerging Role of HMGB1 in the Pathogenesis of Schistosomiasis Liver Fibrosis
Source: Front Immunol. 2018 Sep 12;9:1979. doi: 10.3389/fimmu.2018.01979 (PMC6143665; doi:10.3389/fimmu.2018.01979)
Supplement: Table S1 — Demographic data of patients with schistosomiasis. Sera from schistosomotic patients were kindly donated by Instituto René Rachou—Fiocruz Minas. Sera from healthy donors were collected in a blood bank of the Laboratório Central Noel Nutels (LACEN - Rio de Janeiro), with their agreement for research use. Blood from healthy donors were tested and were negative for HIV and hepatitis. [file Table_1.DOCX]

S1 Table. Demographic data of patients with schistosomiasis

| **Characteristics / Region** | **Gender (%)** | | **Age (mean)** |
| --- | --- | --- | --- |
|  | **Male** | **Female** |  |
| Healthy donors (*n* = 87)  Rio de Janeiro, RJ. | 39.0 | 61.0 | 35.9  range (18-62) |
| Acute schistosomiasis (*n* = 10)  Caju, MG. | 30.0 | 70.0 | 13.9  range (6-34) |
| Chronic schistosomiasis (*n* = 100)  São Pedro, MG. | 45.0 | 55.0 | 26.3  range (3-94) |
