# Supplementary material for: Emerging Role of HMGB1 in the Pathogenesis of Schistosomiasis Liver Fibrosis
Source: Front Immunol. 2018 Sep 12;9:1979. doi: 10.3389/fimmu.2018.01979 (PMC6143665; doi:10.3389/fimmu.2018.01979)
Supplement: Table S2 — Effect of DIC treatment on body weight, relative organs weights, ALT, AST and creatinine in healthy mice. At the end of 56 and 112 days post-treatment, healthy untreated (UT) mice and healthy mice with treated with DIC (DIC) were weighed before sacrifice. Relative liver and spleen weights were calculated as following: Relative organ weight = (absolute weight / body weight) × 100. No significant differences (p > 0.05) were observed with relative liver and spleen weights between the UI and DIC groups. Activities of alanine aminotransferase (ALT), aspartate aminotransferase (AST) and creatinine levels were measured in the sera of UT and DIC healthy mice at days 56 and 112 post-treatment. a ALT values were only significantly different (p < 0.05) between UT and DIC groups at day 112 post-treatment. AST and creatinine values did not show significant differences in both treatments. Data are expressed as the mean ± SD of 20 mice per group. The results were tested by Student's t-test. [file Table_2.DOCX]

S2 Table. Effect of DIC treatment on body weight, relative organs weight, ALT, AST and creatinine in healthy mice.

| **Parameters** | **Time of treatment** | **Groups** | | |
| --- | --- | --- | --- | --- |
|  |  | **UT** | | **DIC** |
| Relative liver weight | 56 days | 4.37 ± 0.49 | 4.35 ± 0.35 | |
|  | 112 days | 5.07 ± 0.12 | 4.94 ± 0.39 | |
| Relative spleen weight | 56 days | 0.61 ± 0.10 | 0.93 ± 0.16 | |
|  | 112 days | 0.35 ± 0.08 | 0.35 ± 0.08 | |
| ALT (mU) | 56 days | 12.44 ± 4.57 | 10.77 ± 4.04 | |
|  | 112 days | 13.17 ± 5.18 | 24.65 ± 5.44^a^ | |
| AST (mU) | 56 days | 32.87 ± 2.88 | 30.71 ± 3.18 | |
|  | 112 days | 27.68 ± 5.07 | 27.68 ± 4.06 | |
| Creatinine (mg/dL) | 56 days | 0.616 ± 0.62 | 0.571 ± 0.26 | |
|  | 112 days | 0.233 ± 0.40 | 0.402 ± 0.14 | |

At the end of 56 and 112 days post-treatment untreated (UT) and treated with DIC (DIC) healthy mice were weighted before sacrifice. Relative liver and spleen weights were calculated as following: Relative organ weight = (absolute weight / body weight) x 100. No significant differences (p>0.05) were observed with relative liver and spleen weights of both UI and DIC groups. Activities of alanine aminotransferase (ALT), aspartate aminotransferase (AST) and creatinine levels were measured in the sera of UT and DIC healthy mice at days 56 and 112 post-treatment. ^a^ALT values were only significantly different (p < 0.05) between UT and DIC groups at day 112 post-treatment. AST and creatinine values did not show significant differences in both treatments. Data are expressed as mean ± SD of 20 mice per group. The results were tested by Student’s t-test.
